# RNA uridyl transferases TUT4/7 differentially regulate miRNA variants depending on the cancer cell type

**DOI:** 10.1261/rna.078976.121

**Published:** 2022-03

**Authors:** Ragini Medhi, Jonathan Price, Giulia Furlan, Beronia Gorges, Alexandra Sapetschnig, Eric A. Miska

**Affiliations:** 1 Department of Genetics, University of Cambridge, Cambridge CB2 3EH, United Kingdom; 2 Wellcome Trust Cancer Research UK Gurdon Institute, University of Cambridge, Cambridge CB2 1QN, United Kingdom; 3 STORM Therapeutics Limited, Moneta Building, Babraham Research Campus, Cambridge CB22 3AT, United Kingdom

**Keywords:** isomiRs, cancer, LIN28A, let-7, miRNA–mRNA, TUT4/7

## Abstract

The human terminal uridyl transferases TUT4 and TUT7 (TUT4/7) catalyze the additions of uridines at the 3′ end of RNAs, including the precursors of the tumor suppressor miRNA let-7 upon recruitment by the oncoprotein LIN28A. As a consequence, let-7 family miRNAs are down-regulated. Disruption of this TUT4/7 activity inhibits tumorigenesis. Hence, targeting TUT4/7 could be a potential anticancer therapy. In this study, we investigate TUT4/7-mediated RNA regulation in two cancer cell lines by establishing catalytic knockout models. Upon TUT4/7 mutation, we observe a significant reduction in miRNA uridylation, which results in defects in cancer cell properties such as cell proliferation and migration. With the loss of TUT4/7-mediated miRNA uridylation, the uridylated miRNA variants are replaced by adenylated isomiRs. Changes in miRNA modification profiles are accompanied by deregulation of expression levels in specific cases. Unlike let-7s, most miRNAs do not depend on LIN28A for TUT4/7-mediated regulation. Additionally, we identify TUT4/7-regulated cell-type-specific miRNA clusters and deregulation in their corresponding mRNA targets. Expression levels of miR-200c-3p and miR-141-3p are regulated by TUT4/7 in a cancer cell-type-specific manner. Subsequently, BCL2, which is a well-established target of miR-200c is up-regulated. Therefore, TUT4/7 loss causes deregulation of miRNA–mRNA networks in a cell-type-specific manner. Understanding of the underlying biology of such cell-type-specific deregulation will be an important aspect of targeting TUT4/7 for potential cancer therapies.

## INTRODUCTION

Endogenous small RNA populations include a distinct class of noncoding RNAs called microRNAs (miRNAs). miRNAs are well-characterized biomolecules with conserved roles in gene silencing. They are typically around 20–23 nt in length with a seed region of two to eight bases near the 5′ end that is able to pair perfectly or imperfectly with 3′ UTRs of target mRNAs (Bartel 2004; Kim et al. 2010; Burroughs and Ando 2019). Upon perfect pairing, miRNAs induce post-transcriptional silencing of the target via endonucleolytic cleavage, while imperfect pairing leads to translational repression (Bartel 2004). Based on conservation of miRNA features (sequence, structure, or function), various miRNAs have been classified under distinct families that are located in different genomic loci (Zou et al. 2014).

Most miRNA loci are transcribed by RNA Pol II and processed at the 5′ and 3′ ends to generate capped and polyadenylated primary precursors of the miRNAs (pri-miRNAs). Subsequently, the RNase III enzyme DROSHA in conjunction with the RNA-binding partner DGCR8, measures ∼22 nt from the terminal loop of pri-miRNAs to introduce a cut and cleaves pri-miRNAs into ∼70 nt pre-miRNA precursors (Kim 2005). Pre-miRNA hairpin precursors undergo further processing by another RNase III enzyme, DICER, to give rise to 5p and 3p mature miRNAs derived either from the 5′ arm or 3′ arm of the hairpin, respectively. The mature miRNA derived from one arm of the pre-miRNA hairpin is preferentially loaded on a functional RISC (RNA-induced silencing complex) and the miRNA derived from the other arm is potentially degraded (Bartel 2018). Additionally, the mature miRNA sequences of a specific miRNA gene are heterogeneous, with differences in the 5′ end or 3′ end (additions or trimming), methylation, SNPs, and ADAR-mediated A-to-I editing (Barturen et al. 2014; Vitsios and Enright 2015) being reported. Such miRNA variants are called isomiRs (Neilsen et al. 2012). Many isomiRs are miRNAs with templated additions (that align with reference sequence) or nontemplated additions (3′ NTA) (Wyman et al. 2011).

With the advent of high-throughput sequencing, diverse 3′ NTA isomiRs have been identified for several miRNAs (Burroughs and Ando 2019). Systematic analysis of the 3′ NTA profiles has revealed that such isomiRs are not experimental artifacts of sequencing techniques but are physiologically relevant (Wyman et al. 2011; Knouf et al. 2013). In addition, such nontemplated additions are conserved across several species (Wyman et al. 2011; Saiselet et al. 2015). Overall, the most abundant modification irrespective of the studied system is addition of uridines (U) or adenines (A) (Chang et al. 2012; Li et al. 2012b; Saiselet et al. 2015). In the nematode *C. elegans*, uridylated miRNAs are higher in abundance than adenylated miRNAs. However, the reverse is true for higher mammals, such as humans and mice (Wyman et al. 2011). Some studies have highlighted that the 3′ NTA profiles change upon viral infection. For example, hepatitis B viral (HBV) infected human liver tissues have lower levels of adenylated miRNAs (Li et al. 2012a). Another study has reported that B cells are enriched in adenylated miRNAs but uridylated isoforms dominate in their secreted exosomes (Koppers-Lalic et al. 2014).

In terms of proportions, mono-A or mono-U additions account for approximately 80% of all NTAs, but the 3′ NTA landscape is not limited to these two. AA, AU, UA, and UU di-nucleotide terminal modifications are also observed and account for ∼1% of addition events (Li 2012a,b). Overall, the abundance of 3′ NTA isomiRs varies depending on the tissue type or disease context and even on the organism of study. Although, at a global level, the proportion of miRNA 3′ NTA is small, a subset of specific miRNAs has a higher proportion (>75%) of isomiRs, which hints toward a biologically important role for their modified counterpart (Yang et al. 2019). Moreover, 3′ NTA additions have been shown to result in miRNA turnover and expression level changes with adenylation conferring stability in contrast to uridylation, which promotes degradation (D'Ambrogio et al. 2012; Heo et al. 2012; Kim et al. 2015; Gutiérrez-Vázquez et al. 2017).

In cancer, microRNA expression levels are useful in different aspects such as diagnosis, determining tumor progression, drug resistance, and the prediction of overall survival. For example, miR-200c and miR-141, both constituting the miR-200c/141 cluster, are diagnostic markers and are up-regulated (Sulaiman et al. 2016) in ovarian cancer (Koutsaki et al. 2017; Chen et al. 2019). These miRNAs mediate the various consequences ranging from increased cell proliferation and metastasis to drug resistance via their target mRNAs (Feng et al. 2014; Sulaiman et al. 2016). Therefore, it is crucial to understand how to modulate and control their expression levels to develop effective anticancer therapeutics.

The terminal RNA uridyl transferases TUT4 and TUT7 (TUT4/7) are the enzymes responsible for catalyzing the addition of Us at the 3′ end of miRNAs such as the tumor suppressor let-7s, to regulate their expression levels (Thornton et al. 2014). TUT4/7 mono-uridylate pre-let-7 miRNAs that have a 1 nucleotide overhang at their 3′ end, thereby restoring the optimal structure for efficient processing by DICER (Heo et al. 2012; Kim et al. 2015). However, in the presence of the oncogenic interactor LIN28A, TUT4/7 dwell longer at a pre-let-7 complex, oligo-uridylate it, and mark it for decay (Yeom et al. 2011; Kim et al. 2015; Faehnle et al. 2017; Wang et al. 2017). In addition, TUT4/7 can uridylate pre-miR-324 (canonical with 2 nt overhang), modulating the DICER cleavage site (Kim et al. 2020). In cancers such as glioblastomas overexpressing TUT4 and TUT7, pre-miR-324 is uridylated at the 3′ end, which leads to the 3′ arm-derived miR-324-3p being more abundant than the miR-342-5p derived from the 5′ arm of the same precursor hairpin (Kim et al. 2020). Importantly, inhibition of TUT4/7-mediated uridylation results in a switch of the strand ratios with the miR-342-5p now being more abundant than the miR-324-3p (Kim et al. 2020). 5p/3p arm switching events have been observed in several independent studies and their ratios have been reported to change in tumor versus normal tissues for several cancer types (Li et al. 2012a,b; Kim et al. 2020). In addition to arm switching, uridylation of miRNAs by TUT4/7 has implications for mRNA target selection. For instance, TUT4/7-mediated di-uridylation has been reported to enable the modified isomiRs to target noncanonical mRNA targets (imperfect seed pairing) via extensive base pairing at the 3′ end, thus expanding the miRNA target repertoire (Yang et al. 2019).

In this study, we explore the TUT4/7-mediated regulation of miRNA variants. We find that TUT4/7 catalyze most of the miRNA uridylation in the prostate cancer cell line DU145 and the ovarian cancer cell line IGROV1. We report that the loss of uridylated isomiRs observed in the TUT4/7 catalytic knockouts (TUT4/7 cKOs) results in a simultaneous gain of adenylated counterparts for specific miRNAs, and that this compensation is not a general phenomenon for all miRNAs. We present examples of miRNAs with no change in expression levels despite a complete switch in isomiR populations. In addition, we identify cell-line or cell-type-dependent and -independent clusters of miRNAs that are deregulated upon TUT4/7 loss. Along with changes in isomiR population and miRNA abundances, we also observe overall slower growth rate and cell-type-specific defects in cell migration in the TUT4/7 cKOs. We also identify specific miRNAs such as miR-200c-3p and miR-141-3p to be regulated by TUT4/7 in a cell-line-specific manner. As miRNAs are increasingly being identified as cancer biomarkers and their expression and sequence profiles can distinguish between normal and tumor tissues (Lu et al. 2005), existence of such competing isomiR populations and TUT4/7-mediated cell-type-specific regulatory networks that control their expression levels hold clues to cancer progression. Thus, our findings provide a new avenue for the development of anticancer therapeutics as well as highlighting the importance of further investigations for the cell-type-specific roles of TUT4/7 in cancer and the underlying isomiR biology.

## RESULTS

### The viable double knockouts of full-length TUT4 and TUT7 show cell-type-specific phenotypes

**FIGURE 1.**
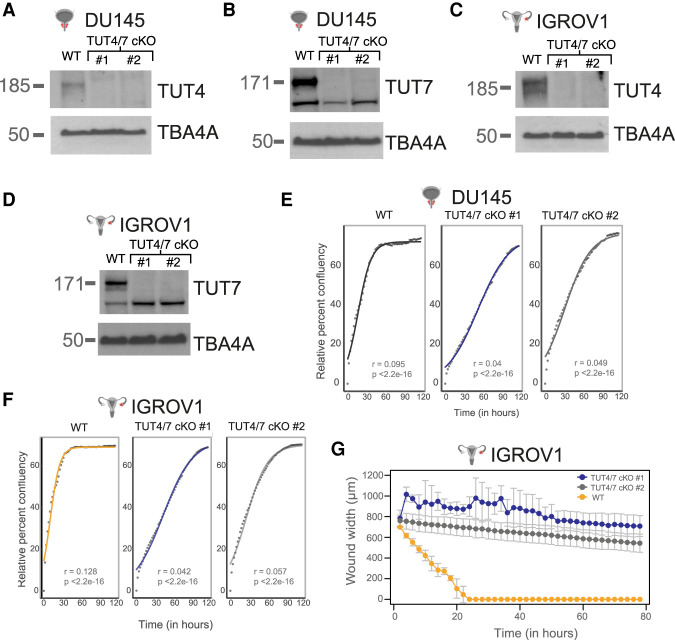
Loss of full-length TUT4 and TUT7 in the TUT4/7 double mutants has a negative impact on cancer cell properties. Molecular masses of TUT4, TUT7, and the loading control α-tubulin (TBA4A) are 185, 171, and 50 kDa, respectively. (*A*,*B*) Western blot images of TUT4 (*A*) and TUT7 (*B*) in independent TUT4/7 double-mutant (cKO) clones (TUT4/7 cKO #1 and TUT4/7 cKO #2) of DU145 (*n* = 3). (*C*,*D*) Western blot images of TUT4 (*C*) and TUT7 (*D*) in independent TUT4/7 double-mutant (cKO) clones (TUT4/7 cKO #1 and TUT4/7 cKO #2) of IGROV1 (*n* = 3). (*E*,*F*) Quantitative cell proliferation assay at 2-h intervals using the Incucyte systems. Solid line indicates curve fitted to the logistic equation with *r* as the growth rate and *p* as the *P*-value of *r*. WT is the control for DU145 (*E*) and IGROV1 (*F*). TUT4/7 cKO #1 and TUT4/7 cKO #2 are the TUT4/7 double mutants. The *y*-axis represents relative percent confluency while the *x*-axis denotes time in hours (*n* = 2). (*G*) Quantitative wound healing assays with measurements at 2-h intervals. The *y*-axis denotes the wound width in µm and the *x*-axis represents time in hours. Cell line = IGROV1. WT is the control IGROV1 cell line. TUT4/7 cKO #1 and TUT4/7 cKO #2 are the TUT4/7 double mutants (*n* = 2).

The RNA uridyl transferases TUT4 and TUT7 are broadly expressed in a diverse range of cell types. This study focuses on the prostate cancer cell line DU145 and the ovarian cancer cell line IGROV1, as both cell lines have been previously reported to show reduced cell proliferation and tumor growth in xenograft models upon TUT4 transcript depletion via siRNAs or shRNAs (Piskounova et al. 2011; Fu et al. 2014). To understand the role of TUT4/7-mediated RNA uridylation in tumorigenesis, we generated double mutants of TUT4 and TUT7 in both cell lines by targeting exon 12 for *TUT4* and exon 14 for *TUT7* upstream of the catalytic domain encoding exon (exon 16 for both TUT4 and TUT7). Western blots probing for TUT4 and TUT7 show that both proteins are endogenously expressed at detectable levels in wild-type cells but are depleted in two independent double mutant clones of both cell lines (Fig. 1A–D), indicating that our CRISPR/Cas9 gene-editing strategy was successful. Contrary to a previous study in cervical-derived HeLa cells, which concluded that the functional activity of both TUT4 and TUT7 is vital for cell viability (Lim et al. 2014), our data show that TUT4 and TUT7 are not required for viability in the prostate cancer cell line DU145 and the ovarian cancer cell line IGROV1. This suggests that the severity of cellular phenotypes upon TUT4/7 loss may be cell-line or tumor specific.

As the TUT4/7 double mutants of DU145 and IGROV1 were viable, we examined the TUT4/7 double mutants for possible defects in cancer cell properties such as cell proliferation, wound healing, and cell migration. We scored for defects in cell proliferation using qualitative colony formation assays followed by Crystal Violet staining and quantitative cell proliferation assays for relative confluency using live-cell imaging at 2-h intervals. Defects in wound healing were also quantified by live-cell imaging at regular intervals after the introduction of a scratch in a >90% confluent well. Impaired cell migration was assessed using transwell migration assays followed by Crystal Violet staining. Compared to the wild-type cells, the TUT4/7 double mutants of DU145 and IGROV1 show a slower cell growth phenotype (Fig. 1E,F; Supplemental Fig. S1A,B). However, the IGROV1 double mutants of TUT4 and TUT7 also show a defect in wound healing unlike the DU145 TUT4/7 double mutants (Fig. 1G; Supplemental Fig. S1C,D). Brightfield images at the start point (day 0) and end point (day 3) of scratch assays show that in the wild-type control, the wound width is reduced to 0 at approximately the 20-h time point, whereas the IGROV1 TUT4/7 double mutants fail to reform the cell monolayer at the end point of the assay (Fig. 1G; Supplemental Fig. S1D).

To investigate whether the defect in wound healing is due to a cell migration defect and hence decoupled from the slow cell growth phenotype, we performed transwell migration assays. Both clones of DU145 TUT4/7 mutants do not show any drastic defect in migration (Supplemental Fig. S1E), while IGROV1 TUT4/7 mutants show slower cell migration compared to wild-type (Supplemental Fig. S1F). Note that cell migration is higher in the metastatic-site-derived DU145 cancer cell line than the ovarian cancer cell line IGROV1 derived from stage III primary solid tumors (Supplemental Fig. S1E,F). Therefore, our results suggest that the loss of the full-length TUT4/7 have negative consequences for cancer cell properties and the severity may depend on the cancer cell line or the stage of cancer progression.

### Loss of full-length TUT4 and TUT7 results in a significant reduction of uridylated miRNAs

To assess the impact of TUT4/7 loss on miRNA populations, we performed small RNA sequencing and explored the modification profile of mature miRNAs. Using Chimira (version 2018; Vitsios and Enright 2015), we first investigated the distribution of miRNA reads with terminal modifications (nontemplated additions, i.e., NTA), A-to-I editing, SNPs, or no modifications in wild-type cells and found that, overall, A-to-I editing and SNPs constituted a small fraction of the total population (<0.2%) (Supplemental Fig. S2A). NTA averaged at ∼7% and ∼12% of the total miRNA reads for IGROV1 and DU145, respectively (Supplemental Fig. S2). The majority of the reads (∼88% for DU145 and ∼93% for IGROV1) belonged to unmodified miRNA variants (Supplemental Fig. S2), which contain canonical miRNAs (exactly matching the mature miRNA template), 3′ trimmed isomiRs (with only the 5′ end matching to the mature miRNA template), 3′ extended isomiRs (no 5′ variation but templated extension in the 3′ end), 5′ trimmed isomiRs (no 5′ variation), 5′ extended isomiRs (no 3′ variation but templated extension in the 5′ end), and multilength variants (both the 5′ and 3′ ends differ from the canonical mature miRNA) (Barturen et al. 2014).

Using sRNAbench (Barturen et al. 2014), we explored the distribution of the aforementioned isomiR variants in the two wild-type cell lines. Both canonical miRNAs (exact) and the 3′ extended isomiRs (lv3pE) constitute the highest fraction with each type occupying ∼36%–38% of the total miRNA reads (Supplemental Fig. S2B). Approximately 8% of the total miRNA population is comprised of the 3′ trimmed isomiRs (lv3pT) (Supplemental Fig. S2B). 5′ extended isomiRs (lv5pE) constitute the lowest fraction (∼0.3%–0.6%) of the unmodified pool (Supplemental Fig. S2B). This fraction is followed by 5′ trimmed isomiRs (lv5pT) and multilength variants (mv) that constitute ∼2%–4% of the total miRNA reads (Supplemental Fig. S2B). Reads with NTA fall in the remaining fraction (others), which is ∼10% and ∼13% of the total miRNA reads in IGROV1 and DU145, respectively (Supplemental Fig. S2B).

We found the 3p mature miRNAs to be more frequently modified than the 5p mature miRNAs in the two cell lines, potentially because of the preexisting 3′ modifications of the precursor hairpins, which also undergo 3′ base additions before DICER processing. For example, TUT4/7 target pre-miRNAs, such as the pre-let-7s, for terminal uridylation (Heo et al. 2012; Kim et al. 2015). Approximately 27% and 29% of the total number of miRNAs have 3p modified isomiRs in the DU145 and IGROV1 cell lines (Supplemental Fig. S2C). Both the cell lines share a high number of unmodified (overlap = 399) and modified (overlap = 167) 3p miRNAs (Supplemental Fig. S2D).

**FIGURE 2.**
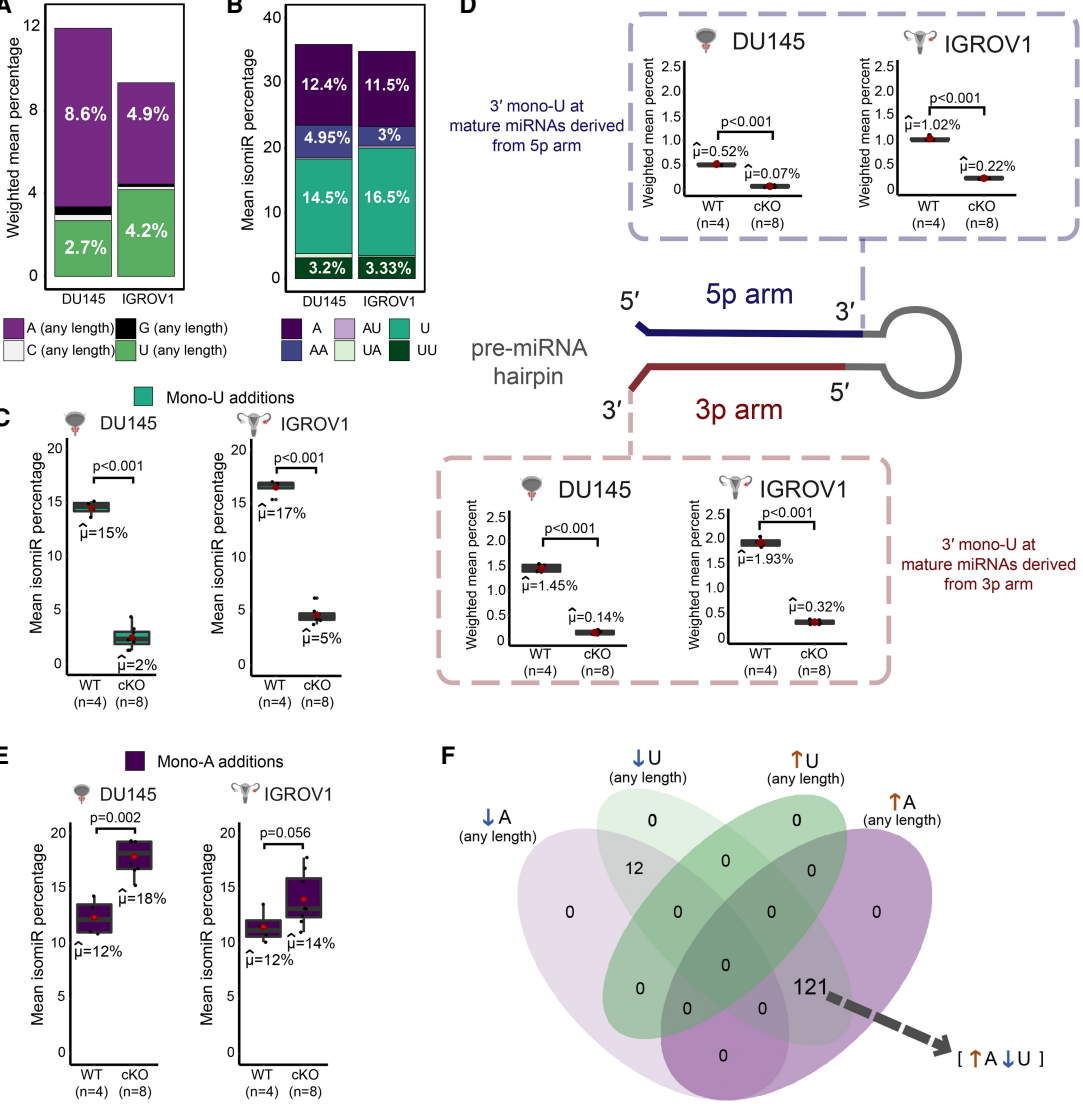
Impact of TUT4/7 loss on proportion of terminally modified miRNA variants. (*A*) “A (any length)”, “C (any length)”, “G (any length)”, and “U (any length)” are nontemplated additions of A, C, G, or U, respectively, with length 1 (in the case of mono additions) to the longest chain of homopolymeric tail detected at the end of an miRNA. (*B*) The NTA types displayed are mono-A additions (A), di-A additions (AA), mono-A addition followed by a mono-U additions (AU), mono-U additions (U), mono-U additions followed by a mono-A additions (UA), and di-U additions (UU). (*C*) Mono-U additions in the wild-type control (WT; *n* = 4) and TUT4/7 double mutants (cKO; *n* = 8) of DU145 and IGROV1. µ cap denotes the mean and *P*-value is indicated by p. (*D*) Mono-U additions at 3p and 5p mature miRNAs in the wild-type control (WT; *n* = 4) and TUT4/7 double mutants (cKO; *n* = 8) of DU145 and IGROV1. µ cap denotes the mean and *P*-value is indicated by p. (*E*) Mono-A additions in the wild-type control (WT; *n* = 4) and TUT4/7 double mutants (cKO; *n* = 8) of DU145 and IGROV1. µ cap denotes the mean and *P*-value is indicated by p. (*F*) Venn diagram showing overlap between miRNAs that show an overall decrease in adenylation (↓A), miRNAs with a decrease in uridylation (↓U), miRNAs with an increase in uridylation (↑U), and miRNAs with an increase in adenylation (↑A).

Next, we explored which one of the nontemplated base additions (A, U, G, or C) is predominantly observed on miRNA 3′ ends. On average, miRNAs with nontemplated G and C additions constitute <0.4% of the total miRNA population (Fig. 2A). Nontemplated A additions represent the highest fraction of all base additions (∼8.6% for DU145 and ∼4.9% for IGROV1), followed by nontemplated U additions or terminal uridylation (∼2.7% for DU145 and ∼4.2% for IGROV1) (Fig. 2A).

Within the nontemplated base modifications, we investigated the mono-A (A), di-A (AA), mono-U (U), di-U (UU), AU, and UA types of terminal additions as they were previously reported to be the most abundant 3′ base modifications (Li et al. 2012a,b). We found AU and UA types of NTA to be the least frequent, with a mean isomiR percentage below 1% (Fig. 2B). Di-A (AA) and di-U (UU) types of NTA are observed at a frequency of ∼3%–5% (Fig. 2B). Single base additions of A and U are the most frequent types of nontemplated additions (Fig. 2B). Mono-uridylation displays a mean isomiR percent of 14.5% and 16.5% in both the DU145 and IGROV1 cell lines, a higher percentage than the one of mono-adenylation in the respective cell lines (Fig. 2B). Therefore, our data show that mono-uridylation and mono-adenylation are the most frequent types of nontemplated additions in the DU145 and IGROV1 cancer cell lines.

Loss of TUT4/7 results in a significant reduction of mono-U additions from isomiR percentages of 15% and 17% in the wild-type (WT) controls to 2% and 5% in the TUT4/7 DU145 and IGROV1 double mutants (Fig. 2C). Although mono-U additions are one of the dominant forms of terminal uridylation, the abundance of U additions of any length at the 3′ end of mature miRNAs is reduced in the TUT4/7 double mutants (Supplemental Fig. S2E). The weighted mean ratio (ratio of number of reads with nontemplated U to the total number of reads) has a significant reduction in the TUT4/7 double mutants of DU145 and IGROV1 compared to the wild-type (Supplemental Fig. S2E). Therefore, this validates that we have generated loss of function mutants of catalytic TUT4/7.

Next, we explored whether TUT4/7 affect mono-U additions at the 3′ end of mature miRNAs derived from both the 3p and the 5p arm of the pre-miRNA hairpin and observed that the loss of TUT4/7 results in significant reduction of mono-U additions in the 3′ terminal end of both 3p and 5p mature miRNAs (Fig. 2D). As 5p miRNAs are uridylated, TUT4/7-mediated uridylation occurs on mature miRNAs post DICER processing. However, mature miRNAs derived from the 3p arm are mono-uridylated at a higher frequency than the mature miRNAs derived from the 5p arm (Fig. 2D). 3p mature miRNAs show a significant reduction in mono-uridylation from 1.45% and 1.93% of weighted mean in the wild-type controls to 0.14% and 0.32% in the TUT4/7 double mutants of DU145 and IGROV1, respectively (Fig. 2D). In comparison to 3p mature miRNAs, 5p mature miRNAs are mono-uridylated at a lower mean frequency of 0.52% and 1.02% in the wild-type control, which significantly reduces to 0.07% and 0.22% in the TUT4/7 double mutants of DU145 and IGROV1, respectively (Fig. 2D). Therefore, mono-uridylation at the 3′ terminal end of 3p mature miRNAs is affected more than on 5p miRNAs upon TUT4/7 loss. However, overall terminal uridylation of any length at the 3′ end of mature miRNAs decreases significantly in the TUT4/7 double mutants of both DU145 and IGROV1.

### Decrease in uridylated isomiRs leads to a simultaneous gain in adenylated counterparts

It has previously been noted that there is an increase in the adenylated proportions of specific miRNAs upon the loss of TUT4/7 (Thornton et al. 2014; Yang et al. 2019; Kim et al. 2020). For example, adenylated reads for miR-324-3p simultaneously increase with loss of uridylated reads in TUT4/7 double knockouts of mouse cells (bone marrow, embryonic fibroblast, embryonic stem cell, and liver) (Kim et al. 2020). In addition, ectopic expression of miR-27a-3p in TUT4/7 double knockouts and wild-type control of HEK293T show that the uridylated fraction of ectopically expressed miR-27a reduces ∼2.5-fold with a concomitant ∼2.3-fold increase of adenylated reads for ectopic miR-27a-3p (Yang et al. 2019). Furthermore, mono-adenylation at 19 predicted targets of TUT4/7, which are all derived from 5p pre-miRNA arms, simultaneously increases upon TUT4/7 depletion and loss of mono-uridylation in the cervical cancer cell line HeLa (Thornton et al. 2014). In line with these previous observations, we observe a concurrent and significant global increase in mono-adenylation with the loss of mono-uridylation in the TUT4/7 double mutants of DU145 and IGROV1 (Fig. 2E). The mean isomiR percentage of mono-adenylation increased from ∼12% in the wild-type controls to ∼18% and ∼14% in the TUT4/7 double mutants of DU145 and IGROV1, respectively (Fig. 2E). This increase in terminal adenylation of mature miRNAs was not limited to the mono-adenylation type of nontemplated A additions, but mature miRNA reads with A additions of any length increased in all the TUT4/7 double mutants (Supplemental Fig. S2F). The increase in adenylated isomiRs was higher in the DU145 TUT4/7 mutants compared to IGROV1 TUT4/7 mutants (Fig. 2E). In contrast to mono-uridylation, mono-adenylation frequency is higher at the terminal ends of 5p mature miRNA compared to 3p mature miRNA (Supplemental Fig. S2G). Mono-adenylation at the 3′ end increased consistently and significantly for both 5p and 3p mature miRNA in the DU145 TUT4/7 mutants relative to the wild-type control (Supplemental Fig. S2G). However, this increase in mono-adenylated isomiRs was observed only in mature miRNA derived from the 3p arm in the TUT4/7 double mutants of IGROV1 compared to the wild-type (Supplemental Fig. S2G). Although there is an increase in mono-adenylation frequency, TUT2, which catalyzes A additions, and the exonuclease PARN, which acts on miRNAs with A addition, do not significantly deregulate at the RNA level (Supplemental Fig. S2H).

**FIGURE 3.**
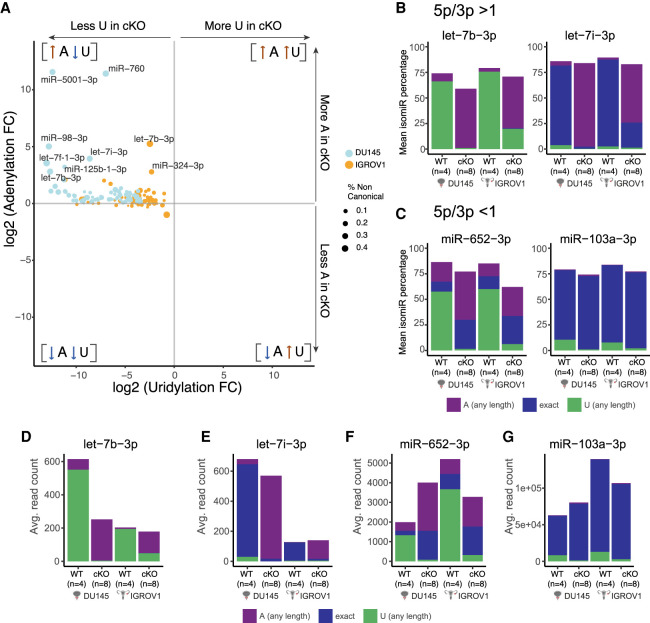
TUT4/7-dependent miRNAs gain adenylation upon loss of uridylation. (*A*) The 133 miRNAs out of the 167 overlapping modified miRNAs in DU145 and IGROV1 are depicted, and each miRNA has a terminal modification frequency of at least >1%. Fold change is calculated in the TUT4/7 double mutants compared to the wild-type control. The *x*-axis denotes logarithmic (base 2) fold-change in uridylation while the *y*-axis displays logarithmic (base 2) fold-change in adenylation in cKO versus WT. The miRNAs specific to IGROV1 are depicted in yellow and miRNAs specific to DU145 are displayed in blue. The labeled miRNAs are the ones with at least fivefold increase in adenylation. (*B*) The *y*-axis indicates mean isomiR percentage. Both let-7b and let-7i have a higher 5p mature miRNA abundance compared to the 3p mature miRNA abundance, that is, a 5p/3p ratio of >1. Proportion of canonical, adenylated, and uridylated isomiRs of let-7b-3p and let-7i-3p is displayed. (*C*) Proportion of canonical, adenylated, and uridylated isomiRs of miR-652-3p and miR-103a-3p with 5p/3p < 1 is displayed. (*D*–*G*) Abundances of 3p mature miRNAs of let-7b-3p (*D*), let-7i-3p (*E*), miR-652-3p (*F*), and miR-103a-2 (*G*) in the WT controls of DU145 and IGROV1 with their corresponding TUT4/7 double mutants (catalytic knockouts) represented by cKOs.

Out of the 167 overlapping modified 3p miRNAs between DU145 and IGROV1 wild-type cells (Supplemental Fig. S2D), 133 miRNAs had at least 1% terminal nontemplated additions. In the TUT4/7 mutants of DU145 and IGROV1, we observed an increase in adenylated isomiRs coupled with a decrease in their uridylated counterparts in 121 miRNAs out of the 133 (Fig. 2F). The miRNAs, let-7b-3p, let-7f-1-3p, let-7i-3p, miR-98-3p, miR-324-3p, miR-760, and miR-5001-3p gain adenylated isomiRs (miRNA reads with A additions of any length) by at least fivefold upon the loss of their uridylated counterparts (miRNA reads with U additions of any length) (Fig. 3A).

Next, we investigated whether NTA modifications are preferentially added to either the 5p or 3p mature sequences of a specific miRNA to mark it for degradation. In our data, let-7b-5p and let-7i-5p are higher in abundance than let-7b-3p and let-7i-3p, respectively, with a ratio of 5p/3p > 1. Both the 3p mature miRNAs of let-7b and let-7i have a higher proportion of modified isomiRs than the 5p miRNAs (Fig. 3B,D,E; Supplemental Fig. S3A,B). Although the canonical let-7i-3p with no terminal modification dominated the total miRNA pool in the wild-type control, the adenylated isomiRs emerged as the dominant isoform in the TUT4/7 double mutants of DU145 and IGROV1 (Fig. 3B). Such up-regulation of adenylated isomiRs upon the loss of uridylated counterparts is not limited to miRNAs with 5p/3p > 1 but is also observed for miRNAs with a higher abundance of 3p miRNAs, that is, 5p/3p < 1. miR-652 with 5p/3p < 1 also shows such changes in modified miRNA population (Fig. 3C,F; Supplemental Fig. S3C). Therefore, the presence of NTA might not be a mark for degradation for 5p/3p strand control. Examples of miRNAs that do not show an increase in adenylated isomiRs include miR-103a-2 (5p/3p < 1). Uridylated miR-103a-3p is significantly reduced in the TUT4/7 catalytic knockouts but adenylated miR-103a-3p does not increase in abundance (Fig. 3C,G; Supplemental Fig. S3D).

Similar to observations made in a recent study (Kim et al. 2020), the 3p mature miRNA of miR-324 was the dominant miRNA in the wild-type controls relative to the 5p mature miRNA and upon TUT4/7 loss, mature miRNAs derived from the 5p arm increased in abundance compared to the 3p mature miRNAs (Supplemental Fig. S3E). This arm switching mechanism is observed in both DU145 and IGROV1 TUT4/7 double mutants (Supplemental Fig. S3E). Thus, miR-324 displays a differential arm preference depending on TUT4/7 expression status. Both the 5p mature miRNA and 3p mature miRNA of miR-324 were marked by the simultaneous increase in adenylation upon the loss of TUT4/7 (Supplemental Fig. S3F). Such TUT4/7-mediated arm switching events were rare in our data set and were not observed in most miRNAs. Supplemental Figure S3G shows an example of an miRNA, miR-103a-2, which does not show arm switching. Thus, we conclude that loss of TUT4/7-mediated uridylation triggers a differential increase in terminal adenylation at the 3′ end for a subset of miRNAs.

### Specific miRNA clusters are deregulated upon the loss of terminal uridylation

**FIGURE 4.**
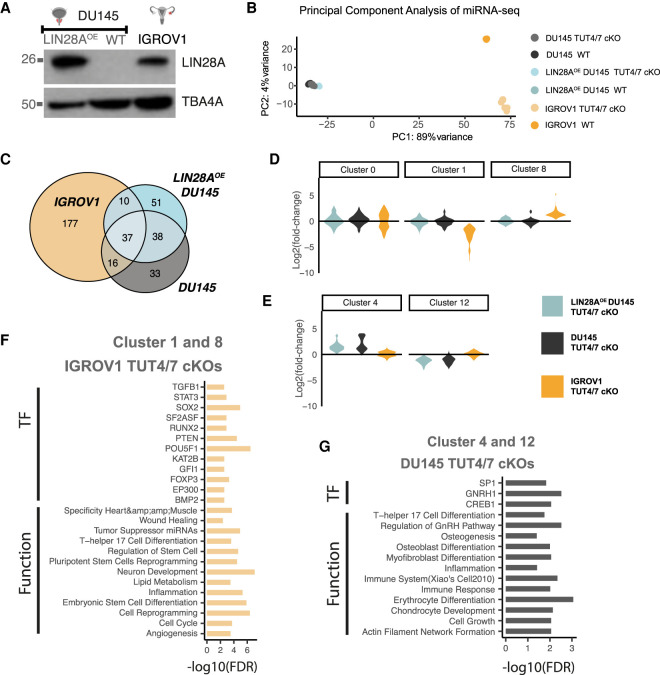
TUT4/7 loss results in cancer cell-type-specific miRNA deregulation. (*A*) Western blot image of LIN28A in the stable cell line LIN28A^OE^ DU145, DU145 (depicted as WT), and IGROV1 with α-tubulin (TBA4A) as a control (*n* = 2). Molecular mass of LIN28A is 26 kDa and of TBA4A is 50 kDa. (*B*) Principal component analysis of miRNA data set. PC1 versus PC2 in miRNA data for all genotypes. (*C*) Venn diagram showing gene overlap. (*D*,*E*) Violin plots displaying the expression trends in Clusters 0, 1, and 8 (*D*) and Clusters 4 and 12 (*E*). Fold change is calculated in the TUT4/7 double mutants (TUT4/7 cKO) relative to their respective wild-type control. (*F*,*G*) miRNA set enrichment analysis was performed with TAM 2.0 (Li et al. 2018). Enriched associations for the miRNA Cluster 1 and Cluster 8 with a similar deregulation pattern in TUT4/7 double mutants of IGROV1 (*F*) and for the miRNA Cluster 4 and Cluster 12 with a similar deregulation pattern in TUT4/7 double mutants of DU145 (*G*).

3′ end uridylation and adenylation of RNAs have been associated with degradation and stability, respectively (D'Ambrogio et al. 2012; Heo et al. 2012; Kim et al. 2015; Gutiérrez-Vázquez et al. 2017). TUT4/7-mediated uridylation has been shown to down-regulate the expression levels of the let-7 family members when TUT4/7 interact with the stemness factor LIN28A (Lehrbach et al. 2009; Piskounova et al. 2011; Wang et al. 2017). Unlike IGROV1, DU145 cells do not express detectable LIN28A protein levels (Fig. 4A). To explore the TUT4/7-mediated regulation of miRNA expression levels, we generated stable DU145 wild-type and DU145 TUT4/7 double mutant cell lines overexpressing lentiviral integrated CMV-driven LIN28A cDNA copies lacking introns and the 3′ UTR (henceforth referred to as LIN28A^OE^ DU145) and analyzed their small RNA transcriptome (Fig. 4A). A principal component analysis (PCA) shows that the first principal component (PC1) explains the majority (89%) of the variation in the data set, which appears to be due to differences in the cell line type (Fig. 4B), while the second principal component (PC2) separates IGROV1 TUT4/7 mutants from the wild-type controls (Fig. 4B).

A PCA on all genotypes of the prostate cancer DU145-derived cell lines shows that PC1 versus PC2 captures clonal variation, whereas PC2 versus PC3 captures the variation in the data that is associated with the presence or absence of TUT4/7 (Supplemental Fig. S4A). A higher number of miRNAs were deregulated in the IGROV1-derived mutants relative to the DU145-derived TUT4/7 catalytic knockouts (Fig. 4C; Supplemental Fig. S4B). Therefore, the absence of TUT4/7-mediated miRNA regulation has a higher impact on the IGROV1 cell line relative to the prostate cancer cell line DU145.

Next, we explored the effect of loss of uridylation on the expression levels of let-7 family members in a LIN28A-dependent context. We divided the let-7 family into the following classes: (i) canonical Group I miRNAs with 2 nucleotide overhangs at the 3′ end (1), (ii) noncanonical Group II miRNAs with 1 nucleotide overhang at the 3′ end (2), (iii) CSD^+^ let-7s, which contains the (U)GAU-binding motif for the cold shock domain (CSD) of LIN28A (+), and (iv) CSD^−^ let-7s, which do not contain the sequence motif for LIN28A binding via its CSD domain (−).

In DU145, all let-7 miRNAs are generally either down-regulated or unchanged in TUT4/7 cKOs (Supplemental Fig. S4C). Based on previous data (Ustianenko et al. 2018), miRNA from CSD^+^ precursors should be up-regulated in LIN28A-positive TUT4/7 cKOs. This trend is true for all CSD^+^ let-7 miRNAs except miR-98 in DU145-derived TUT4/7 cKOs (LIN28A- -positive vs. LIN28A negative) (Supplemental Fig. S4C). However, we do not observe the same trend of up-regulation for CSD^+^ let-7s in IGROV1 TUT4/7 catalytic knockouts, which endogenously express LIN28A (Supplemental Fig. S4C). Hence, this suggests that TUT4/7-mediated let-7 regulation is cell-line specific and does not always depend on LIN28A in a cellular environment, contrary to what is known from in vitro tailing reactions (Piskounova et al. 2011; Thornton et al. 2012; Kim et al. 2015).

Next, to identify miRNAs that depend on LIN28A-TUT4/7 for the regulation of their expression, we performed *k*-means clustering (*k* = 15) of all miRNAs excluding let-7 family members and identified 14 miRNA clusters (Supplemental Fig. S4D). Cluster 0 (number of miRNAs, *N* = 60) consists of miRNAs whose expression does not significantly change (Fig. 4D). Cluster 1 contains miRNAs (*N* = 76) that are down-regulated and Cluster 8 miRNAs (*N* = 34) that are up-regulated only in the IGROV1 TUT4/7 cKOs (Fig. 4D). Similarly, Cluster 4 represents miRNAs (*N* = 32) that are up-regulated and Cluster 12 miRNAs (*N* = 9) that are down-regulated only in the DU145-derived TUT4/7 cKOs (Fig. 4E). On performing miRNA set enrichment analysis using TAM 2.0 (Li et al. 2018), we found that only IGROV1 TUT4/7 cKO-specific clusters (Cluster 1 and 8) display “wound healing” as an enriched function (Fig. 4F), suggesting that deregulation of the miRNAs in these two clusters results in defective wound healing in the IGROV1 TUT4/7 double mutants. The miRNA set enrichment analysis for Cluster 4 and 12 show “cell growth” and “differentiation”-related terms, suggesting that these miRNAs might contribute to the slow growth phenotype in the TUT4/7 double mutants of the metastatic-site derived DU145 cancer cell line (Fig. 4G). In addition, miRNAs belonging to Cluster 2 (*N* = 19) and Cluster 3 (*N* = 81) show a general trend of down-regulation and up-regulation, respectively, independent of the cell line, with “differentiation” emerging as the most enriched function (Supplemental Fig. S4E,F). Therefore, TUT4/7-mediated regulation of miRNA expression levels is miRNA specific, and subsets of miRNAs are regulated in a cell-type-specific manner.

### Cell-type-specific negative correlation of miRNA–mRNA interactions upon functional loss of TUT4/7 activity

miRNAs mediate gene silencing by targeting mRNAs post-transcriptionally. Hence, global deregulation of miRNA abundance may severely impact the mRNA transcriptome. To assess the effects of TUT4/7 loss on the transcriptome, we sequenced poly(A)-selected transcripts in our samples. PCA analyses on our mRNA-seq data sets were similar to those performed on our miRNA data sets (Supplemental Fig. S5A–C). According to the current model of TUT4/7-mediated mRNA regulation (Lim et al. 2014), TUT4/7 are considered general mRNA decay factors and global up-regulation of mRNA is expected upon the loss of uridylation. However, we did not observe this global trend of up-regulation in the DU145 and IGROV1 TUT4/7 cKOs (Supplemental Fig. S5D). In addition, minimal overlap of deregulated mRNAs between the cell lines suggests that TUT4/7 differentially regulate mRNAs depending on the cell line (Supplemental Fig. S5E). Upon gene-ontology analysis of the deregulated mRNAs, “immune response” pathways were enriched in the prostate DU145 TUT4/7 cKOs (Supplemental Fig. S5F). This is not unusual given the role of TUT4/7 in antiviral processes and transposable element regulation (Le Pen et al. 2018; Warkocki et al. 2018). In comparison, a higher number of processes relating to “wound healing” and “cell migration” were enriched in the ovarian IGROV1 TUT4/7 cKOs (Supplemental Fig. S5F–H).

**FIGURE 5.**
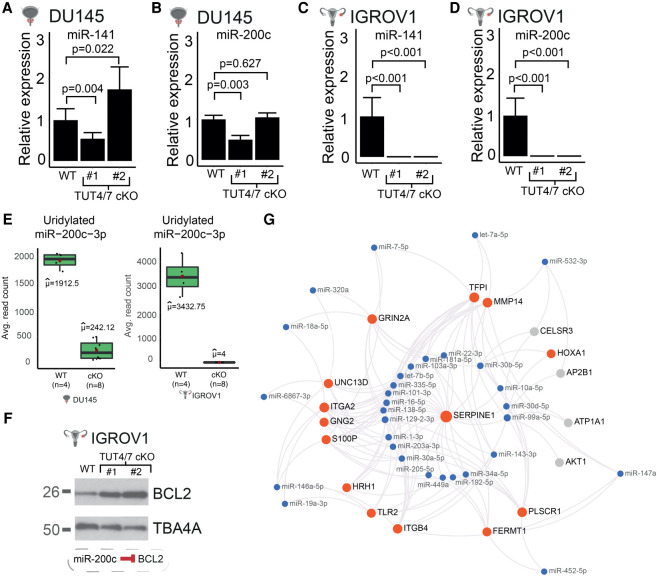
IGROV1-specific miRNA–mRNA interactions. (*A*,*B*) RT-qPCR quantification of miR-141-3p (*A*) and miR-200c-3p (*B*) in DU145 and derived TUT4/7 cKOs. Number of biological replicates (*n*) for WT = 4. TUT4/7 cKO #1 represents one independent clone (*n* = 4) and TUT4/7 cKO #2 (*n* = 3) represents the other. *P*-values are denoted by p and are determined by Student's *t*-test. (*C*,*D*) RT-qPCR quantification of miR-141-3p (*C*) and miR-200c-3p (*D*) in IGROV1 and derived TUT4/7 cKOs. Number of biological replicates (*n*) for WT = 4. TUT4/7 cKO #1 represents one independent clone (*n* = 4) and TUT4/7 cKO #2 (*n* = 4) represents the other. *P*-values are denoted by p and are determined by Student's *t*-test. (*E*) Abundances of uridylated isomiRs of miR-200c-3p in DU145, IGROV1, and derived TUT4/7 cKOs from RNA-seq data analyzed with sRNAbench software (Barturen et al. 2014). (*F*) Down-regulation of miR-200c in TUT4/7 cKOs is accompanied by up-regulation of one of its targets, BCL2. (*G*) Each miRNA (in blue) is connected to mRNA targets that are implicated in cell migration or wound healing. Red dots denote mRNAs that are down-regulated, otherwise the mRNA is denoted in gray. This network was created using miRNet (Fan et al. 2016).

Unlike the DU145 cell line derived from a metastatic site, the IGROV1 cell line is originally derived from a primary tumor in Stage III, which is before the metastatic Stage IV (Stone et al. 1978; Bénard et al. 1985). During metastasis, the metastasis-associated miR-200c cluster is up-regulated and it is also used as diagnostic markers in ovarian cancer (Sulaiman et al. 2016; Chen et al. 2019). We found that miR-200c-3p and miR-141-3p belonging to the miR-200c cluster are targets of TUT4/7 and are regulated in a cell-line-specific manner (Fig. 5A–E; Supplemental Fig. S6A,B and Supplemental Fig. S6D,E). Both miRNAs are repressed only in the IGROV1 TUT4/7 cKOs (Fig. 5A–D; Supplemental Fig. S6A,B and Supplemental Fig. S6D,E). In turn, the well-characterized target of miR-200c, the antiapoptotic factor BCL2 (Feng et al. 2014) is now up-regulated (Fig. 5F; Supplemental Fig. S6C). Although BCL2 has elevated expression levels in many cancers and is an anticancer target, overexpression of BCL2 alone in cells does not impart tumorigenic potential but rather can have prosurvival benefits (Lu et al. 1995; Kirkin et al. 2004; Czabotar et al. 2014).

Additionally, we used miRNet 2.0 (Chang et al. 2020) to identify miRNA–mRNA interactions related to wound healing and cell migration in the IGROV1-derived TUT4/7 cKOs. We used the list of IGROV1-specific down-regulated mRNAs and up-regulated miRNAs as queries, and gene ontology terms related to wound healing or cell migration were selected. Subsequently, the miRNA–mRNA interaction network in Figure 5G was obtained. Many cancer-cell invasion and migration-promoting genes such as *SERPINE1*, *PLSCR1*, *ITGA2*, *MMP14*, etc. are significantly down-regulated in the IGROV1 cells (Fig. 5G). According to our network, these mRNAs interact with up-regulated miRNAs such as those belonging to the miR-30 family (miR-30a-5p and miR-30b-5p) and miR-99a-5p (Fig. 5G). Although such negative correlation of expression levels of miRNA and their mRNA targets is widely observed in this study, we also observe several examples where a negative correlation is not observed. For example, despite the trend of down-regulation in their mRNA targets (Fig. 5G), let-7a-5p and let-7b-5p expression levels do not drastically change. In addition, both miR-7-5p and its targets are down-regulated in the IGROV1 TUT4/7 cKOs, thus showing a positive correlation with its targets. Such observations could be due to multiple miRNAs having shared targets.

In summary, TUT4/7 are master regulators and versatile in their RNA regulation function. TUT4/7-mediated miRNA regulation depends on the cell line of study. This leads us to hypothesize that certain, still unknown, cell-type-specific upstream factors exert overall influence over TUT4/7 recruitment. This will require further investigations as our data suggests that the severity of TUT4/7 inhibition may differ depending on the cancer cell type or the stage of cancer progression. Thus, a better understanding of cell-type-specific interactors of TUT4/7 will be crucial in stratifying tumors for potential treatment with TUT4/7 inhibitors.

## DISCUSSION

TUT4/7-mediated RNA metabolism is complex and shapes the transcriptomic network in a cell-type-dependent manner. Here, we explored the global TUT4/7-mediated RNA regulatory networks in two different cancer cell line models with distinct tissues of origin. The two cell lines used are the prostate cancer cell line DU145, which is derived from a metastatic site and has a high stem cell–like population and the cell line IGROV1 derived from the primary solid tumor of stage III ovarian cancer (Stone et al. 1978; Bénard et al. 1985; Pfeiffer and Schalken 2010). We found that TUT4/7 catalytic knockouts display an overall slower cell proliferation, but with cell-line-specific differences in cell-migration phenotypes (Fig. 1E–G; Supplemental Fig. S1A–F). Such cell-line-specific phenotypic defects could be due to loss of TUT4/7-mediated 3′ uridylation of specific miRNAs (Fig. 2C; Supplemental Fig. S2E). Based on *k*-means clustering, the deregulated miRNAs can be divided into groups that follow the same trend of deregulation in the TUT4/7 catalytic knockouts (Supplemental Fig. S4D). These groups can be further classified into distinct miRNA clusters that are either cell-type-independent or -dependent (Fig. 4D,E; Supplemental Fig. S4E). TUT4/7 loss has a greater impact on the cancer cell properties of the ovarian cancer cell line IGROV1 than the prostate cancer cell line DU145. GO terms relating to cell migration are exclusively limited to the IGROV1-specific miRNA clusters, whereas terms relating to differentiation emerge in the DU145-specific clusters (Fig. 4F,G; Supplemental Fig. S4F). In addition, we identify deregulated mRNA targets relating to cell migration exclusively in the ovarian cancer cell line (Supplemental Fig. S5F–H). The miRNAs miR-200c-3p and miR-141-3p are regulated by TUT4/7 in a cell-type-dependent manner (Fig. 5A–E; Supplemental Fig. S6A–E). Therefore, our results suggest that TUT4/7-dependent miRNA-mediated mRNA regulation varies depending on the cancer cell line of study, which results in observed differences in defects of cancer cell properties. These inherent differences in TUT4/7-mediated RNA regulation could also possibly depend on the cancer status and the stage of cancer progression, where TUT4/7 modulate transcriptomic changes based on the current requirements for tumorigenesis. Our data suggests that in DU145, TUT4/7-mediated miRNA regulation may help in maintaining an enriched population of stem cell–like cells as the removal of TUT4/7 function results in the emergence of “differentiation”-related GO terms (Fig. 4G). In addition, for the IGROV1 cell line, TUT4/7-mediated regulation may prepare the tumor for the next stage of cancer, that is, metastasis, as cell migration, “wound healing,” and “angiogenesis” related genes are deregulated upon TUT4/7 loss (Fig. 4F; Supplemental Fig. S5G).

We also find that the regulation of the tumor suppressor let-7 family members, which is known to be dependent on the LIN28A-TUT4/7 pathway, does not strictly adhere to the previously postulated uniform down-regulation model inside cells, and that LIN28A-TUT4/7-mediated regulation depends on the cell line of study (Supplemental Fig. S4C). Similar to observations made in a recent study (Ustianenko et al. 2018), this is contrary to the uniform down-regulation model observed in vitro (Heo et al. 2008, 2009; Viswanathan et al. 2008). Moreover, we find that, beyond let-7, miRNAs do not generally show a trend in expression level change that depends on the LIN28A-TUT4/7 pathway (Supplemental Fig. S4D).

The loss of catalytic activity of TUT4/7 leads to an increase in adenylated miRNAs. This has been observed previously upon the depletion of TUT4/7 but was limited to a few miRNA examples (Thornton et al. 2014; Yang et al. 2019; Kim et al. 2020). Here we provide a global view of changes in miRNA variant populations in the TUT4/7 double mutants of the prostate cancer cell line DU145 and the ovarian cancer cell line IGROV1 (Fig. 2A–F; Supplemental Fig. S2A–G). Many effector enzymes similar to TUT4/7 such as TUT2, TENT4A, and TENT4B have been identified and shown to catalyze A additions. Upon the loss of TUT4/7, as specific miRNAs undergo increased adenylation, it is possible that adenylating enzymes such as TUT2 and TENT4A/B are in competition with TUT4/7 for the same miRNA substrate. Therefore, in the absence of TUT4/7, the adenylating enzymes can now access these specific miRNAs and target them for adenylation as we observe an increase in adenylation of TUT4/7-dependent miRNAs in our data (Fig. 2E; Supplemental Fig. S2F,G).

Additionally, reads for isomiRs with nontemplated base additions were considered to be artifacts of sequencing technologies, but this notion was subsequently dismissed and invalidated (Wyman et al. 2011; Knouf et al. 2013). One of the critical factors that proves their relevance in biological processes is the association of NTA-modified isomiRs with active components of RNA-induced silencing complexes (RISCs) (Burroughs et al. 2010; Yang et al. 2019). Argonaute (AGO) proteins are responsible for loading miRNAs into RISC complexes. Existence of multiple AGO proteins may indicate selective and preferential sorting of miRNAs into individual AGOs. Indeed, miRNAs terminally modified with adenine additions have reduced association with AGO2 and AGO3 (Burroughs et al. 2010). High base complementarity between miRNA:mRNA is a factor for intrinsic endonuclease activity of AGO2, and adenine additions might impact its slicer activity (Liu et al. 2004; Song et al. 2004; Burroughs et al. 2010). However, other terminal base modifications did not show any significant changes in AGO associations (Burroughs et al. 2010). Therefore, the change in isomiR population for specific miRNAs in the TUT4/7 catalytic knockouts might also affect preferential AGO loading. The underlying consequences might need further investigation.

Another two important aspects of 3′-modified isomiRs are miRNA turnover and target regulation. Terminal oligo-uridylation of miRNAs has been associated with instability, whereas adenylation is attributed to increased stability of miRNAs (D'Ambrogio et al. 2012; Heo et al. 2012; Kim et al. 2015; Gutiérrez-Vázquez et al. 2017). However, insignificant global changes in miRNA half-lives indicated that isomiR dynamics might not control miRNA expression levels as a general universal mechanism (Kingston and Bartel 2019). In line with previous studies, control of miRNA turnover for specific miRNAs including the let-7 family members by TUT4/7-mediated 3′ uridylation is hard to refute and rule out (Heo et al. 2012; Thornton et al. 2014; Kim et al. 2015). Moreover, NTA isomiRs such as the uridylated fraction have been shown to regulate noncanonical target repertoires and to increase the diversity of targets for a certain miRNA by mono- or di-nucleotide changes in its 3′ terminus (Yang et al. 2019). Furthermore, half-lives of miRNA are longer than their mRNA targets, with a median half-life of 25 h in proliferating mouse embryonic fibroblasts (Kingston and Bartel 2019). Therefore, NTA modifications of readily available stable miRNAs might be a means of fast response to the changing stimuli through targeting diverse repertoires of noncanonical mRNA targets. This probably provides a greater control on the overall physiological fate.

With this new knowledge of TUT4/7 biology, it is apparent that TUT4/7 are master regulators of the global transcriptome. Due to its involvement in the LIN28A:let-7 axis, special attention has been brought on the efficacy of targeting TUT4 and TUT7 to negatively impact cancer proliferation and progression (Piskounova et al. 2011; Lin and Gregory 2015; Kim et al. 2020). Based on our findings, it is important to consider that inhibition of these uridylating enzymes comes together with compensatory and a wide range of transcriptomic changes that vary depending on the cancer cell type or status. Therefore, further understanding of and exploring such dynamics of gene regulation is crucial to avoid nonspecific deleterious side effects when targeting TUT4/7 for anticancer therapeutics.

## MATERIALS AND METHODS

### Cell culture

Prostate cancer cell line DU145 (ATCC/LGC, Cat. #HTB-81, Lot#61761869) and the ovarian cancer cell line IGROV1 (NCI via Charles River Laboratories) were selected as the cell lines for use as in vitro models. DU145 complete media was made with the following reagents: EMEM (Gibco, Cat. #41090093), 1× NEAA (Gibco, Cat. #11140035), 10% FBS (Gibco, Cat. #10270106, Lot #42G6287K), 1× Antibiotics (Life Technologies, Cat. #15140122), and 5 mL Sodium Pyruvate (Life Technologies, Cat. #11360-039). IGROV1 complete media was comprised of the following: RPMI-1640 (Gibco, Cat. #72400054), 1× NEAA (Gibco, Cat. #11140035), 10% FBS (Gibco, Cat. #10270106; Lot #42G6287K), 1× Antibiotics (Life Technologies, Cat. #15140122). Cell lines were maintained at 37°C, 5% CO_2_ in a humid incubator. Mycoplasma testing was performed using an EZ-PCR Mycoplasma Test Kit (Geneflow, Cat. #K1-0210) according to the manufacturer's recommendation. At 80%–90% confluency, adherent cell cultures were dissociated using 1× Trypsin-EDTA (Life Technologies, Cat. #25300054) and passaged at a 1:4 dilution. Icons to represent different tissues were created from BioRender.com.

### Plasmid preparation and cloning

One shot TOP10 chemically competent cells (Invitrogen, Cat. #C4040-03) were used and incubated at 37°C overnight post-transformation according to the manufacturer's recommendation. Following this, 4 mL of LB media was inoculated with a single colony and incubated at 37°C for 24 h. Purification and extraction of plasmids for genotyping was performed using a PureLink Quick Plasmid MiniPrep Kit (Invitrogen, Cat. #K210001). Plasmid sequences and their identity were validated by Sanger sequencing (Genewiz Services) or using restriction enzymes. Endotoxin free plasmid preparation for transfection into cells was performed using a Qiagen Plasmid Maxi Kit (Cat. #12163). Restriction enzymes required for cloning into the desired plasmid site were identified using CLC Workbench software. Successful digestions were confirmed using agarose gel electrophoresis. One percentage of agarose gel was prepared using 1 g of agarose powder (Bioline, Cat. #BIO-41025) in 100 mL of 1× TBE buffer (Tris-borate-EDTA) and 1 µL of ethidium bromide solution (Merck, Cat. #E1510). The digested products and 1 kb Plus DNA Ladder (Life Technologies, Cat. #10787018) were loaded on separate wells of the agarose gel and run at a constant voltage of ∼130 V for 40 min with 1× TBE as the buffer. Digested linear plasmid at the correct band size was cut and recovered from the gel using a Zymoclean Gel DNA Recovery Kit (Zymo Research, Cat. #D4002). Purity of the extracted DNA was checked using the 260 to 280 nm and the 260 to 230 nm wavelength absorbance ratios. If the ratio of absorbance at 260 to 230 nm was lower than 2.0, then a second round of purification was performed using a PureLink PCR Purification Kit (Life Technologies, Cat. #K310002). Ligations at different vector/insert ratios were done overnight at 20°C using a T4 DNA Ligase (NEB, Cat. #M0202T).

### Generation of catalytic knockouts

The protocol described in the following section is an adaptation of the method described in Chiang et al. (2016). Risk assessments and training prior to performing the gene-editing experiments were completed and approved (Reference No. EM68).

*TUT4 (ZCCHC11)* exon 12 and *TUT7 (ZCCHC6)* exon 14 were successfully targeted using CRISPR/Cas9-mediated gene editing. Cas9 expressing plasmid All-in-One nickase vector (Chiang et al. 2016) was used as the backbone vector (kind gift by Steve Jackson laboratory, Gurdon Institute). This is an All-in-One vector that expresses a Cas9 mutant nickase (RuvC^D10A^), enhanced green fluorescent proteins (EGFP), and the target-specific small guide RNAs. Two pairs of guide RNAs were ligated with T4 DNA ligase (NEB, Cat. #M0202T), after restriction digestion of the vector using Bsa1 (NEB, Cat. #R0535S) and BbsI (NEB, Cat. #R0539S) as per NEB recommendations. One shot TOP10 chemically competent cells (Invitrogen, Cat. #C4040-03) were transformed and selected on LB plates with ampicillin selection. The transformed *E. coli* was grown in LB broth with ampicillin, and the recombinant plasmids were purified using a Qiagen Plasmid Maxi Kit (Cat. #12163). Plasmids were sent for Sanger sequencing (Genewiz Services) to further validate the ligation and sequence of the sgRNA.

A total of 8 µg of endotoxin free plasmids were aliquoted in 30 µL of water. 10^7^ cells were resuspended in 1 mL of resuspension buffer R (Neon Transfection System 100 µL Kit, Thermo Scientific, Cat. #MPK10025). An amount of 120 µL of cells in resuspension buffer were added to the plasmid mix. The DNA-cell mix was then electroporated in 3 mL of electrolyte buffer E2 (Neon Transfection System 100 µL Kit, Thermo Scientific, Cat. #MPK10025) at 1400 V for 30 msec at the rate of 1 pulse. The cells were immediately transferred to a serum-rich media. EGFP expression after 48 h of electroporation denoted successful transfection.

Cells were trypsinized and washed with phosphate buffered saline (PBS). Centrifugation was done at 1000 rpm for 3 min to pellet the cells. The cell pellet was resuspended in 500 µL of PBS. EGFP-positive cells were either sorted in a single-cell suspension per well of a 96-well plate or bulk sorted in a single tube with serum-rich media. Bulk sorted control cells and EGFP-positive cells were plated in a 10 cm Petri dish with serum-rich media. Crude cell lysates were prepared using 5 µL of cell suspension in 20 µL of DirectPCR Lysis reagent containing Proteinase K (Viagen Biotech, Cat. #302-C) by following manufacturer instructions. Primers were designed either ∼300 bp or ∼600 bp away from the site targeted by the sgRNAs. PCR was performed using Q5 high fidelity polymerase (NEB, Cat. #M0491) using recommended settings. Clones with unexpected band shifts as compared to the wild-type were then sent for Sanger sequencing to confirm deletions and insertions. Genomic DNA for genotyping was purified using a DNeasy Blood & Tissue Kit (Qiagen, Cat. #69504). Mutations on each copy of the gene were further explored using a TOPO TA Cloning Kit (Invitrogen, Cat. #450030) according to the manufacturer's recommendations. Further validation was done via western blotting

### Stable cell line generation

The Lenti-X 293T cell line was kindly provided by the Steve Jackson laboratory (Gurdon Institute). A risk assessment (Reference No. EM67) was completed and approved before performing experiments that used lentiviruses. psPAX2 (Addgene Plasmid #12260) that expresses gag and pol genes along with pMD2.G (Addgene Plasmid #12259) that expresses the VSV-G envelope protein were used as packaging plasmids. Validation of psPAX2 was done with EcoRI (NEB, Cat. #R3101), which results in two bands at ∼6 kb and ∼4 kb on agarose gel electrophoresis. Validation of pMD2.G was done by double digestion with BamH1 (NEB, Cat. #R3136) and HindIII (NEB, Cat. #R3104), which results in two bands at ∼3 kb and ∼2.8 kb on agarose gel electrophoresis. All transfer vectors were driven by the CMV promoter and had puromycin as the antibiotic selective marker.

The protocol for lentiviral production was obtained from the Steve Jackson laboratory (Gurdon Institute). For virus production, 10 cm Petri dishes (Corning, Cat. #10212951) were precoated with 0.1% gelatin for 15 min. The Lenti-X 293T cells were seeded at 1:3 and 1:4 dilutions on two gelatin-coated plates. The following day, the cell cultures at 80%–90% confluency were chosen for lentiviral production. 7.23 µg of psPAX2, 1.56 µg of pMD2.G, and 2.93 µg of the transfer plasmid with the transgene of interest were aliquoted in a 1.5 mL Eppendorf tube and thoroughly mixed by pipetting (Tube 1). An amount of 624 µL of Opti-MEM (Gibco, Cat. #11058021) and 35.1 µL of TransIT-Lenti transfection reagent (Mirus Bio, Cat. #MIR6600) were aliquoted in another 1.5 mL Eppendorf tube and thoroughly mixed (Tube 2). Both Tube 1 and Tube 2 were incubated for 5 min at room temperature in a biosafety cabinet in a containment level 2 room. Subsequently, the contents of Tube 2 were added and mixed with Tube 1 and incubated for 25–30 min at room temperature. After the incubation, the Lenti-X 293T cells were transfected with the mix and lentiviruses from the condition media were collected after 48 h. The collected viruses were syringed through a 0.45 µm filter (Sartorius, Cat. #16555-K). The harvested lentiviruses were then stored at 4°C for up to a week or at −70°C for longer storage.

Recipient cells were passaged at 1:2, 1:3, and 1:4 dilution. Cell cultures at 60%–70% confluency were chosen for transduction. Cells were initially transduced with a range of viral doses in fivefold increments starting at 10 µL. The maximum viral dose was ∼500 µL per well. Complete media was used as the dilution media. Transduced cells were incubated for 48 h.

Puromycin (InvivoGen, Cat. #ant-pr-1) was used as the antibiotic selective marker. Prior to transduction, the optimal puromycin dose was determined by incubating cells at 0, 0.5, 1, 1.5, 2, and 4 µg/mL of puromycin diluted in complete media. Cells were incubated for a period of 10 d with media replacement every 3–4 d. The minimum puromycin concentration that was lethal for 100% of the noninfected cells was selected as the optimal puromycin selection concentration for that cell line. After transduction, infected cells were trypsinized and seeded in a new well of a six-well plate along with a noninfected control in a separate well and incubated with the optimal puromycin dose for 10 d with media replacement every 3–4 d. The nontransduced control did not survive after 10 d but the cells with a successful integration event survived the puromycin selection and treatment. Stable cell lines were further validated for the presence of the transgene on a western blot or under a fluorescence microscope if the transfer plasmid had an EGFP or mCherry marker.

### Western blot

Precast NuPAGE 3%–8% Tris-acetate gels (Invitrogen, Cat. #EA0378BOX) and NuPAGE 4%–12% Bis-Tris Gel (Invitrogen, Cat. #NP0335BOX) were used for separating the proteins as per manufacturer instructions. Running buffers, NuPAGE TA SDS (Invitrogen, Cat. #LA0041), NuPAGE MOPS SDS (Invitrogen, Cat. #NP0001), and NuPAGE Transfer buffer (Invitrogen, Cat. #NP00061) were used. Primary antibodies used were ZCCHC11 (Proteintech, Cat. #18980-1-AP, Lot#00010451), ZCCHC6 (Proteintech, Cat. #25196—1-AP), ZCCHC6 (Merck, Cat. #HPA020620), LIN28A (Cell Signaling, Cat. #3978), LIN28B (Cell Signaling, Cat. #4196), and Tubulin (Merck, Cat. #9026). Secondary antibodies used were anti-rabbit (GE Healthcare, Cat. #NA934) and anti-mouse (GE Healthcare, Cat. #NA931).

### RNA extraction and quality control

RNA was isolated using TRIsure (Bioline, Cat. #BIO-38033) and phase lock tubes (Quantabio, Cat. #733-2478) following standard chloroform-isopropanol extraction. Isolated RNA then underwent TURBO DNase treatment (Ambion, Cat. #AM1907) to remove DNA contaminants. Purity values were measured using Nanodrop. Qubit RNA BR Assay Kit (Thermo Fisher Scientific, Cat. #Q10210) was used to quantify RNA. Reverse transcription was performed using appropriate TaqMan kits (Applied Biosystems) and qPCR was done as per instructions. Analysis of the qPCR results and statistics were performed using the R package “pcr” (Ahmed and Kim 2018).

### Small RNA library preparation and analysis

The small RNA libraries were prepared using a NEXTFlex Small RNA-seq Kit v3 (Cat. #NOVA-5132-06) according to the manufacturer's recommendations. The 3′ adaptors, TGGAATTCTCGGGTGCCAAGG were trimmed using cutadapt (Martin 2011) and further processed to trim the first and last four random bases that act as unique molecular identifiers or UMIs. Use of such UMIs ensures the identification of PCR duplicates that arise as a result of PCR amplification steps. Quality checks were performed using FastQC (Andrews 2010) to determine whether miRNAs had a clear peak at the expected size. Small RNA reads were analyzed with miRDeep2 (default parameters; Friedländer et al. 2012) using the *H. sapiens* (hg38) reference genome. Counts were imported into R and differential expression analysis was performed with DESeq2 (FDR < 0.01; Love et al. 2014). Principal component analysis (PCA) in R was performed to determine whether samples of the same genotype clustered together. *K*-means clustering (*k* = 15) of miRNAs was performed to group miRNAs with similar expression patterns across the samples. miRNA set analysis was performed with TAM 2.0 (Li et al. 2018). For proportion of modifications such as SNPs, analysis was performed using Chimira software (Vitsios and Enright 2015) and counts were obtained. All subsequent analyses of the modification profiles and counts were done using sRNAbench software (Barturen et al. 2014). Ratios or percentages of nontemplated additions were calculated by dividing the modified reads by the total number of reads (unless specified otherwise). Statistical analysis was performed using the ggstatsplot package in R (Patil 2018).

### mRNA library preparation and analysis

The mRNA sequencing libraries were prepared using NEBNext Ultra II Directional RNA Library Prep Kits (NEB, Cat. #E7765S) with the oligo dT selection module according to the manufacturer's recommendation. First, raw reads were trimmed for adaptors, low quality sequences, and short reads with Trimmomatic (parameters: ILLUMINACLIP: TruSeq3-SE.fa:2:30:10 SLIDING-WINDOW:4:28 MINLEN:20; Bolger et al. 2014). For the second step, ribosomal RNAs were removed with sortmeRNA (default parameters; Kopylova et al. 2012). Following this, read mapping was done to the reference genome *H. sapiens* (hg38) with HISAT2 (default parameters; Kim et al. 2015), and reads were then counted on genes with HTSeq-count (Anders et al. 2015). Counts were imported into R and differential gene expression analysis was performed with DESeq2 (FDR < 0.01; Love et al. 2014). Gene ontology analysis of differentially expressed genes was performed with g:Profiler (Raudvere et al. 2019). Further, statistical analyses on normalized counts for the boxplots were performed using the ggstatsplot package in R (Patil 2018). The web-based platform miRNet 2.0 (Chang et al. 2020) was used to identify miRNA–mRNA interactions. The list of IGROV1-specific down-regulated mRNAs and up-regulated miRNAs or vice versa were used as query, and gene ontology terms related to wound healing or cell migration were selected under function explorer (algorithm- hypergeometric test; database- GO:BP).

### Cell proliferation assays

Several dilutions of the DU145 wild-type and mutant cell lines (200, 400, 600, 800, 1000, and 1200 cells/well) were seeded in a six-well plate. Plates were incubated at 37°C and 5% CO_2_ in a humid incubator for 15 d. For IGROV1, 50,000/10,000 cells/well in a 24-well plate were seeded and incubated for 4 d. At the end point, plates were washed and stained with Crystal Violet (Merck, Cat. #V5265-250ML). In addition, proliferation assays at different dilutions and plate formats were performed using a Sartorius/Essen Biosciences IncuCyte System. For the 96-well plate format, the cell dilutions for each well per genotype were 10,000, 20,000, or 30,000 in technical replicates of three. Confluency was determined by label-free time lapse imaging at 2-h intervals using the IncuCyte System. Relative percent confluency at each time point was calculated by subtracting the initial confluency of seeded cells at the start point (time = 0) from the confluency at each time point.

### Growth curve measurements

Raw proliferation data (percent confluency) was obtained from time-lapse imaging using the IncuCyte analysis software. Data were analyzed using the growthcurver R package (Sprouffske and Wagner 2016). Initial confluency was subtracted from the confluency value at each time point. The growth rate was obtained by fitting the data to a logistic equation as given below (Sprouffske and Wagner 2016).

$$N_{t}=K/(1+e^{-rt}((K-N_{0})/N_{0})$$

In this logistic equation, *N*_0_ and *N*_*t*_ are cell confluency at the beginning and at time *t*, respectively. *K* is the maximum carrying capacity and *r* is the growth rate.

### Scratch assay

Cells were seeded in IncuCyte ImageLock 96-well plates (Sartorius, Cat. # 4806). At 90%–100% confluency, a uniform scratch was introduced using the IncuCyte Cell Migration Kit (Sartorius, Cat. #4493) and following manufacturer's instructions. Wound closure was measured by time lapse imaging at 2-h intervals and results were analyzed using the IncuCyte system. The R code for analyzing data was obtained from Grada et al. (2017).

### Transwell migration assay

8.0 µm pore 6.5 mm Corning Transwell inserts in a 24-well format (Merck, Cat. #CLS3422-48EA) were used for a qualitative cell migration assay. For the experimental group, the lower chamber comprised of 800 µL of cell culture medium supplemented with 10% fetal bovine serum (FBS) (Life Technologies, Cat. #10270106, Lot #42G6287K). For the control group, cell culture media without FBS was pipetted into the lower chamber. Adherent cells were trypsinized with 1× Trypsin (Life Technologies, Cat. # 25300054) and harvested in media without FBS. 150 μl of cell dilutions with 5 × 10^5^ cells/mL for DU145 were pipetted carefully into the upper insert chamber. For IGROV1, 100,000–150,000 cells were pipetted into the upper insert chamber. Transwell plates were incubated for 24 h at 37°C and 5% CO_2_ in a humid incubator. At the end point, plates were washed twice in 1× PBS and stained with Crystal Violet (Merck, Cat. #V5265-250ML) for 10 min. After staining, inserts were washed with water. The nonmigrated cells on the inside of the insert were removed with a cotton swab. In summary, this protocol was adapted from an application note titled “Cell migration and invasion quantification assay with acetic acid-dependent elution of Crystal Violet” by Corning, Life Sciences, Shanghai, China.

## DATA DEPOSITION

The data sets and code generated during this study are available at NCBI's Gene Expression Omnibus and are accessible through GEO Series accession number GSE179741.

## SUPPLEMENTAL MATERIAL

Supplemental material is available for this article.

## COMPETING INTEREST STATEMENT

E.A.M. is a founder of STORM Therapeutics Limited, Cambridge, UK and A.S. and B.G. are full-time employees of STORM Therapeutics Limited, Cambridge, UK. R.M. is a PhD student funded by STORM Therapeutics Limited, Cambridge, UK.

## Supplementary Material

Supplemental Material

## Figures and Tables

**Ragini Medhi:**
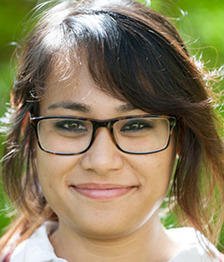


## MEET THE FIRST AUTHOR

***Meet the First Author(s)* is a new editorial feature within *RNA*, in which the first author(s) of research-based papers in each issue have the opportunity to introduce themselves and their work to readers of *RNA* and the RNA research community. Ragini Medhi is the first author of this paper, “RNA uridyl transferases TUT4/7 differentially regulate miRNA variants depending on the cancer cell type.” Ragini obtained her PhD in 2020 from the Gurdon Institute, University of Cambridge, focusing on terminal RNA modifications in cancer, with a view toward understanding novel anticancer therapeutics.**

**What are the major results described in your paper and how do they impact this branch of the field?**

We found that the terminal uridyl transferases, TUT4/7, are not essential for viability in two cancer cell lines of distinct tissue origins. This is the first successful creation of loss-of-function mutants of both TUT4 and TUT7 in the two cell lines, DU145 (prostate cancer) and IGROV1 (ovarian cancer). In addition, we show global transcriptomic changes with a simultaneous increase of adenylated miRNAs upon the loss of the uridylated counterparts. Furthermore, we provide examples of miRNAs that are regulated by TUT4/7 differently in each cell type. Last, severity of phenotypes upon TUT4/7 loss depends on the cancer cell type. Moreover, our paper provides a number of unique resources including loss-of-function TUT4/7 mutants and associated sequencing data to unravel the complex TUT4/7 biology in cancer. In addition, we highlight the importance of differences of TUT4/7 mechanisms in distinct cancer types, which is informative for disease-positioning hypotheses in developing cancer therapies.

**What led you to study RNA or this aspect of RNA science?**

I was fascinated by RNA interference and microRNAs when I learnt about it as an undergraduate. I actively sought research opportunities to study RNA biology. Luckily, soon enough I got the opportunity to do some real science in the RNA field during my Masters program. Here, I did research on small RNA pathways for germline maintenance in the nematode *C. elegans*. Ever since, RNA biology has continued to captivate me.

**During the course of these experiments, were there any surprising results or particular difficulties that altered your thinking and subsequent focus?**

The most difficult moment was unfortunately not related to the actual research but the COVID-19 pandemic and the challenges it brought. We had to restructure and streamline our research focus for the paper due to this.

**Are there specific individuals or groups who have influenced your philosophy or approach to science?**

My parents are my biggest inspirations who kickstarted my interest in science. Both being chemistry professors, they always nurtured curiosity and fact-driven scientific questioning as I grew up.

I would like to dedicate this paper to my father, Professor Okhil Medhi, who sadly passed away in February 2021 owing to a long-term illness. He always had cool stories about the inventors and scientists behind the inventions and discoveries that I really enjoyed as a kid and also as an adult.
