# Supplementary material for: RNA uridyl transferases TUT4/7 differentially regulate miRNA variants depending on the cancer cell type
Source: RNA. 2022 Mar;28(3):353–70. doi: 10.1261/rna.078976.121 (PMC8848932; doi:10.1261/rna.078976.121)
Supplement: Supplemental Material [file supp_28_3_353__DC1.html]

RNA uridyl transferases TUT4/7 differentially regulate miRNA variants depending on the cancer cell-type — RNA uridyl transferases TUT4/7 differentially regulate miRNA variants depending on the cancer cell type — Supplemental Material 

# RNA uridyl transferases TUT4/7 differentially regulate miRNA variants depending on the cancer cell type

## Supplemental Material

- Supplemental\_material\_.pdf
