# Supplementary material for: RNA uridyl transferases TUT4/7 differentially regulate miRNA variants depending on the cancer cell type
Source: RNA. 2022 Mar;28(3):353–70. doi: 10.1261/rna.078976.121 (PMC8848932; doi:10.1261/rna.078976.121)
Supplement: Supplemental Material [file supp_078976.121_Supplemental_material_.pdf]

Supplementary Material for:

"RNA uridyl transferases TUT4/7 differentially regulate miRNA variants depending on the cancer cell-type"

Ragini Medhi[1,2], Jonathan Price[2], Giulia Furlan[2], Beronia Gorges[3], Alexandra Sapetschnig[3], Eric A. Miska[1,2,*]

[1]Department of Genetics, University of Cambridge, Downing Street, Cambridge CB2 3EH, UK

[2]Wellcome Trust Cancer Research UK Gurdon Institute, University of Cambridge, Tennis Court Road, Cambridge CB2 1QN, UK

[3]STORM Therapeutics Limited, Moneta Building, Babraham Research Campus, Cambridge, CB22 3AT, UK

*Corresponding author: eam29@cam.ac.uk

SUPPLEMENTARY FIGURE LEGENDS

Figure S1: Related to Figure 1. Cell proliferation and migration properties of TUT4/7 double mutants. (A) Crystal violet staining at the end of a colony formation assay. Number of cells at the start = 400. End day number = 15; number of replicates, n=3; TUT4/7 cKO #1 is one independent TUT4/7 double mutant clone and TUT4/7 cKO #2 is the other. WT denotes the wildtype control. (B) Crystal violet staining at the end of cell proliferation assay. End day number = 4; number of replicates, n = 2; TUT4/7 cKO #1 is one independent TUT4/7 knockout clone and TUT4/7 cKO #2 is the other. WT denotes the wildtype control. (C) Quantitative wound healing assays. The y-axis denotes the wound width in µm, and the x-axis represents time in hours (n=2). Cell line = DU145. WT is the control DU145 cell line. TUT4/7 cKO #1 and TUT4/7 cKO #2 are the TUT4/7 double mutants. (D) Brightfield images of TUT4/7 double mutants (TUT4/7 cKO) of IGROV1 and the wildtype control (WT) on day 0 (start point) and day 3 (end point) of the scratch assay. (E and F) Transwell cell migration assay for DU145 TUT4/7 double mutants (E) and IGROV1 TUT4/7 double mutants (F) with WT as their respective controls. TUT4/7 cKO #1 and TUT4/7 cKO #2 are the TUT4/7 double mutants (n=2).

Figure S2: Related to Figure 2. Proportion of modified isomiRs in DU145-derived and IGROV1-derived cell lines. (A) WT#1, WT#2, WT#3 and WT#4 are the four biological replicates of either DU145 denoted by the grey line or of IGROV1 denoted by the yellow line. Weighted mean proportion is the ratio of unmodified or modified reads specific to the type of modification (A to I, SNP or NTA) to the total number of reads mapping to miRNAs in the sample. (B) The miRNA population is dominated by miRNAs with variations in their 3′ end. Canonical miRNAs which exactly match their template are represented by “exact”; miRNAs with 3′ templated extensions are denoted by “lv3pE”; miRNAs with a trimmed 3′ end are indicated by “lv3pT”. Similarly, miRNAs with 5′ templated extensions are denoted by “lv5pE” and miRNAs with a trimmed 5′ end are indicated by “lv5pT”. “mv” denotes multi-length variants which do not match both the 5′ end and 3′ end. The rest of the miRNAs are placed in “others”. (C) Bar chart with the number of modified and unmodified 3p mature miRNAs for DU145 and IGROV1 on the y-axis. (D) Venn diagram showing overlaps of modified and unmodified 3p mature miRNAs between the two cell lines. (E) Non-templated U-

additions of any length in the wildtype control (WT; n=4) and TUT4/7 double mutants (cKO; n=8) of DU145 and IGROV1. µ cap denotes the mean and the statistics used are described at the bottom of each boxplot. (F) Non-templated A-additions of any length in the wildtype control (WT; n=4) and TUT4/7 double mutants (cKO; n=8) of DU145 and IGROV1. µ cap denotes the mean and the statistics used are described at the bottom of each boxplot. (G) Mono-A additions at 3p and 5p mature miRNAs in the wildtype control (WT; n=4) and TUT4/7 double mutants (cKO; n=8) of DU145 and IGROV1. µ cap denotes the mean and p-value is indicated by p. (H) The y-axis indicates normalised counts. The expression counts depict changes in the exonucleases PARN along with the adenylating TENT, TUT2 in the prostate cancer cell line DU145. WT = Wildtype control (n=4) and cKO = TUT4/7 double mutants (n=8). p-value (p) is denoted at the bottom of each plot. µ cap indicates the mean.

Figure S3: Related to Figure 3. TUT4/7-mediated variations in isomiR populations in select miRNAs. (A) Proportion and abundances of canonical, adenylated and uridylated isomiRs of let-7b-5p. (B) Proportion and abundances of canonical, adenylated and uridylated isomiRs of let-7i-5p. (C) Proportion and abundances of canonical, adenylated and uridylated isomiRs of miR-652-5p. (D) Proportion and abundances of canonical, adenylated and uridylated isomiRs of miR-103a-2-5p. (E) 5p/3p arm switching of miR-324 in the WT and TUT4/7 double mutants (cKOs) in DU145 and IGROV1. (F) Proportion of canonical, adenylated and uridylated isomiRs of miR-324-3p and miR-324-5p. (G) Abundances of 5p and 3p mature miRNAs of miR-103a-2 in the prostate cancer cell line DU145 (WT) and its corresponding TUT4/7 double mutants (cKOs).

Figure S4: Related to Figure 4. Analysis of the miRNA deregulation in TUT4/7 double mutants relative to the control. (A) Principal component analysis of miRNA dataset. PC1 versus PC2 and PC2 versus PC3 in miRNA data for DU145 prostate cancer cell line and related genotypes. The two different clones of the same genotype are highlighted by blue and grey circles. (B) Global miRNA deregulation trend with LIN28A^{OE} DU145 in blue, DU145 in grey and IGROV1 in yellow. (C) Heatmap representing expression levels of the let-7 family members in the TUT4/7 double mutant clones (TUT4/7 cKO #1 and TUT4/7 cKO #2) and the wildtype controls (WT) of LIN28A^{OE} DU145, DU145 and IGROV1. Each column represents a biological

replicate. The let-7 miRNAs belonging to Group I are denoted by "1" and those belonging to Group II are denoted by "2". "+" indicates that the precursor of the let-7 miRNA contains the (U)GAU sequence motif for LIN28A binding via its CSD domain and "-" indicates the absence of this binding motif. The colour key on the left indicates the z-score or scaled normalised expression from high (in brown) to low (in blue). (D) K-means (k=15) clustering of all miRNAs except the let-7 family groups the miRNAs into 14 distinct groups with similar expression patterns. The TUT4/7 double mutant clones are represented by TUT4/7 cKO #1 and TUT4/7 cKO #2 while the wildtype control of LIN28A^{OE} DU145, DU145 and IGROV1 is denoted by WT. Each column represents a biological replicate. The colour key on the left indicates the z-score or scaled normalised expression from high (in brown) to low (in blue). (E) Violin plots displaying the expression trends in Clusters 2 and 3. Fold change is calculated in the TUT4/7 double mutants (TUT4/7 cKO) relative to their respective wildtype control. (F) miRNA set enrichment analysis was performed with TAM 2.0 (Li et al., 2018). Enriched associations for the miRNA Cluster 2 and Cluster 3 with a similar deregulation pattern in TUT4/7 double mutants independent of the cell line of origin.

Figure S5: Related to Figure 5. Deregulated mRNAs in TUT4/7 double mutants. (A, B and C) Principal component analysis of mRNA dataset. PC1 versus PC2 in mRNA data for all genotypes(A), PC1 versus PC2 in mRNA data for DU145 prostate cancer cell line and related genotypes (B). The two different clones of the same genotype are highlighted by blue and grey circles. PC2 versus PC3 in mRNA data for the DU145 prostate cancer cell line and related genotypes (C). (D) Global mRNA deregulation trend with LIN28A^{OE} DU145 in blue, DU145 in grey and IGROV1 in yellow. (E) Venn diagram showing gene overlap. (F, G and H) Gene Ontology analysis of differentially expressed genes was performed with g:Profiler (Raudvere et al., 2019). Enriched terms for the differentially regulated mRNAs in DU145 TUT4/7 double mutants (F). Enriched terms for the differentially regulated mRNAs in IGROV1 TUT4/7 double mutants (G). Enriched terms for the differentially regulated mRNAs in LIN28A^{OE} DU145 TUT4/7 double mutants (H).

Figure S6: Related to Figure 5. miR-200c cluster and BCL2 in TUT4/7 double mutants. (A) Expression levels of miR-141-3p in DU145, LIN28A^{OE} DU145, IGROV1 and derived TUT4/7 cKOs from RNA-seq data analysed with miRDeep2 (Friedländer

et al., 2012). Number of biological replicates (n) for WT = 4. TUT4/7 cKO #1 represents one independent clone (n=4) and TUT4/7 cKO #2 (n=4) represents the other. (B) Expression levels of miR-200c-3p in DU145, LIN28A^{OE} DU145, IGROV1 and derived TUT4/7 cKOs from RNA-seq data analysed with miRDeep2 (Friedländer et al., 2012). Number of biological replicates (n) for WT = 4. TUT4/7 cKO #1 represents one independent clone (n=4) and TUT4/7 cKO #2 (n=4) represents the other. (C) Expression levels of BCL2 in DU145, LIN28A^{OE} DU145, IGROV1 and derived TUT4/7 cKOs from RNA-seq data. Number of biological replicates (n) for WT = 4. TUT4/7 cKO #1 represents one independent clone (n=4) and TUT4/7 cKO #2 (n=4) represents the other. (D) Abundances of canonical, adenylated and uridylated isomiRs of miR-141-3p in DU145, IGROV1 and derived TUT4/7 cKOs from RNA-seq data analysed with sRNAbench software (Barturen et al., 2014). (E) Abundances of canonical, adenylated and uridylated isomiRs of miR-200c-3p in DU145, IGROV1 and derived TUT4/7 cKOs from RNA-seq data analysed with sRNAbench software (Barturen et al., 2014).

Figure S7: Raw western blots. (A) Raw western blot images relating to Figures 1A-D. (B) Raw western blot images relating to Figure 4A. (C) Raw western blot image for BCL2 for Figure 5F. (D) Raw western blot image for TBA4A for Figure 5F.

Figure S1

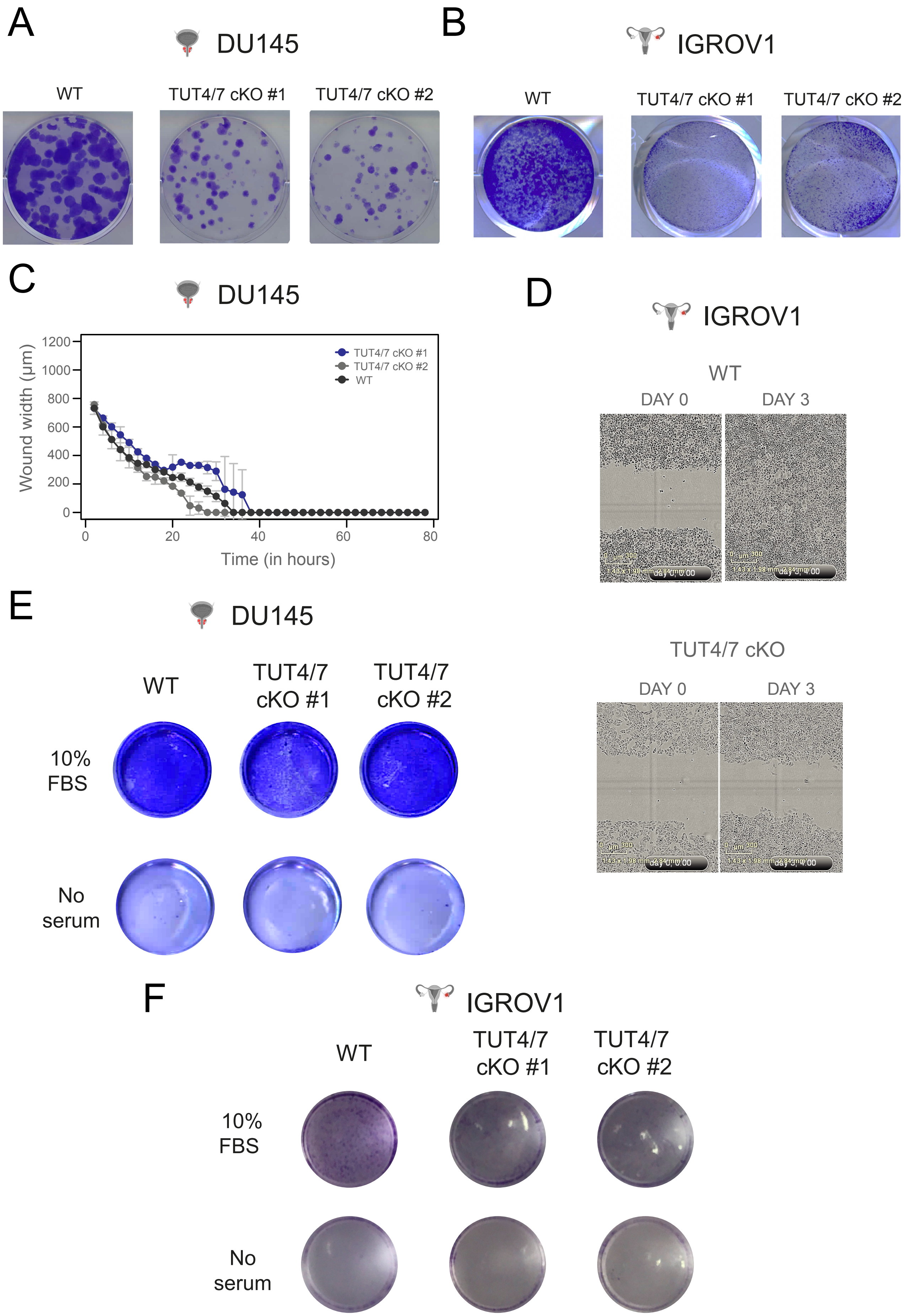

A
DU145
WT
TUT4/7 cKO #1
TUT4/7 cKO #2
B
IGROV1
WT
TUT4/7 cKO #1
TUT4/7 cKO #2
C
DU145
TUT4/7 cKO #1
TUT4/7 cKO #2
WT
Wound width (μm)
1200
1000
800
600
400
200
0
0
20
40
60
80
Time (in hours)
D
IGROV1
WT
DAY 0
DAY 3
TUT4/7 cKO
DAY 0
DAY 3
E
DU145
WT
TUT4/7 cKO #1
TUT4/7 cKO #2
10% FBS
No serum
F
IGROV1
WT
TUT4/7 cKO #1
TUT4/7 cKO #2
10% FBS
No serum

Figure S2

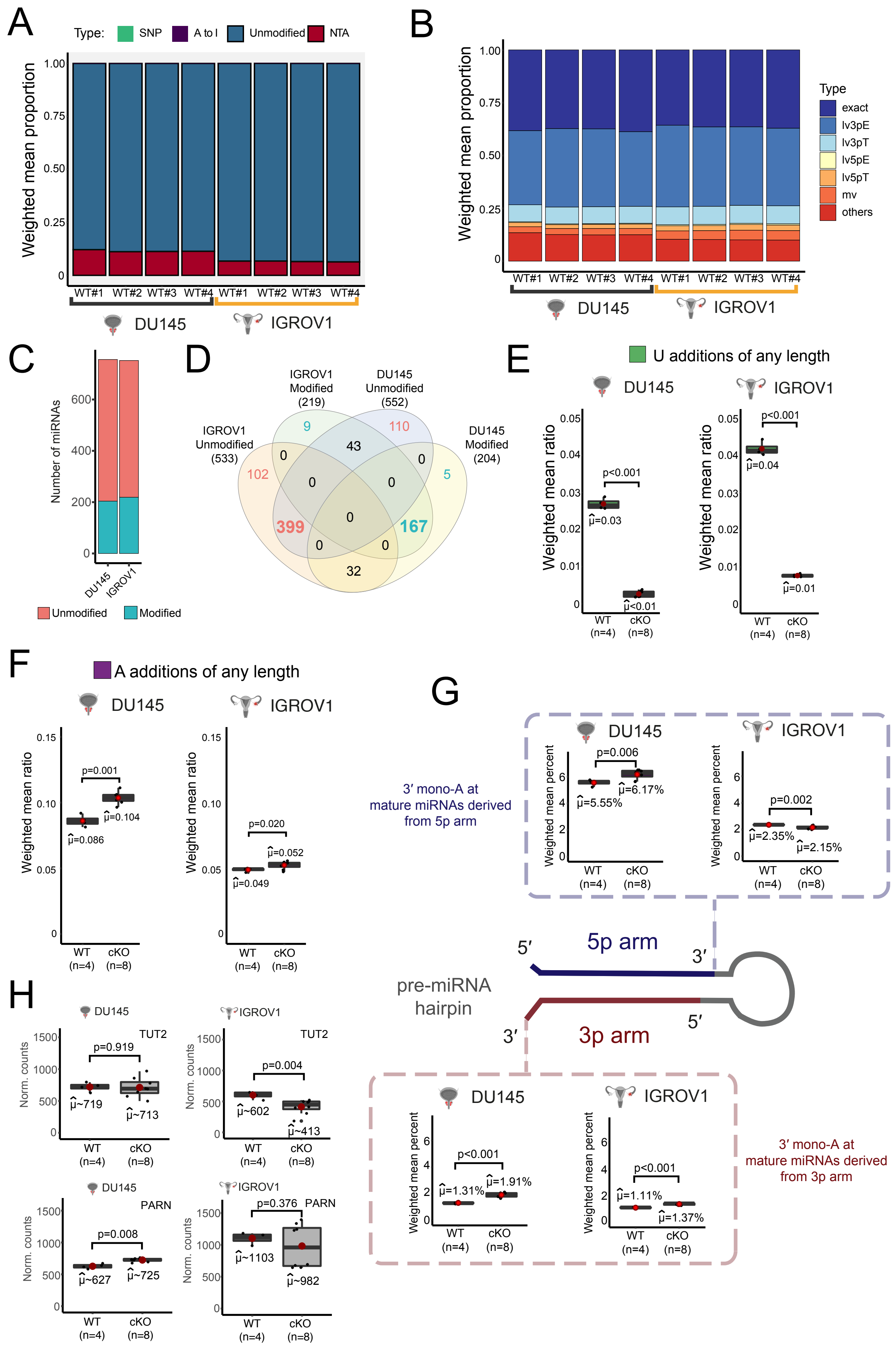

A
Type: SNP A to I Unmodified NTA
Weighted mean proportion
WT#1 WT#2 WT#3 WT#4 WT#1 WT#2 WT#3 WT#4
DU145
IGROV1
B
Weighted mean proportion
Type
exact
Iv3pE
Iv3pT
Iv5pE
Iv5pT
mv
others
C
Number of miRNAs
DU145
IGROV1
Unmodified
Modified
D
IGROV1 Modified (219)
DU145 Unmodified (552)
IGROV1 Unmodified (533)
DU145 Modified (204)
399
167
E
U additions of any length
Weighted mean ratio
p<0.001
μ̂=0.03
μ̂<0.01
μ̂=0.04
μ̂=0.01
WT (n=4)
cKO (n=8)
F
A additions of any length
p=0.001
μ̂=0.086
μ̂=0.104
p=0.020
μ̂=0.049
μ̂=0.052
G
3′ mono-A at mature miRNAs derived from 5p arm
Weighted mean percent
p=0.006
μ̂=5.55%
μ̂=6.17%
p=0.002
μ̂=2.35%
μ̂=2.15%
5p arm
3p arm
pre-miRNA hairpin
5′
3′
p<0.001
μ̂=1.31%
μ̂=1.91%
μ̂=1.11%
μ̂=1.37%
3′ mono-A at mature miRNAs derived from 3p arm
H
Norm. counts
TUT2
p=0.919
μ̂~719
μ̂~713
p=0.004
μ̂~602
μ̂~413
PARN
p=0.008
μ̂~627
μ̂~725
p=0.376
μ̂~1103
μ̂~982

Figure S3

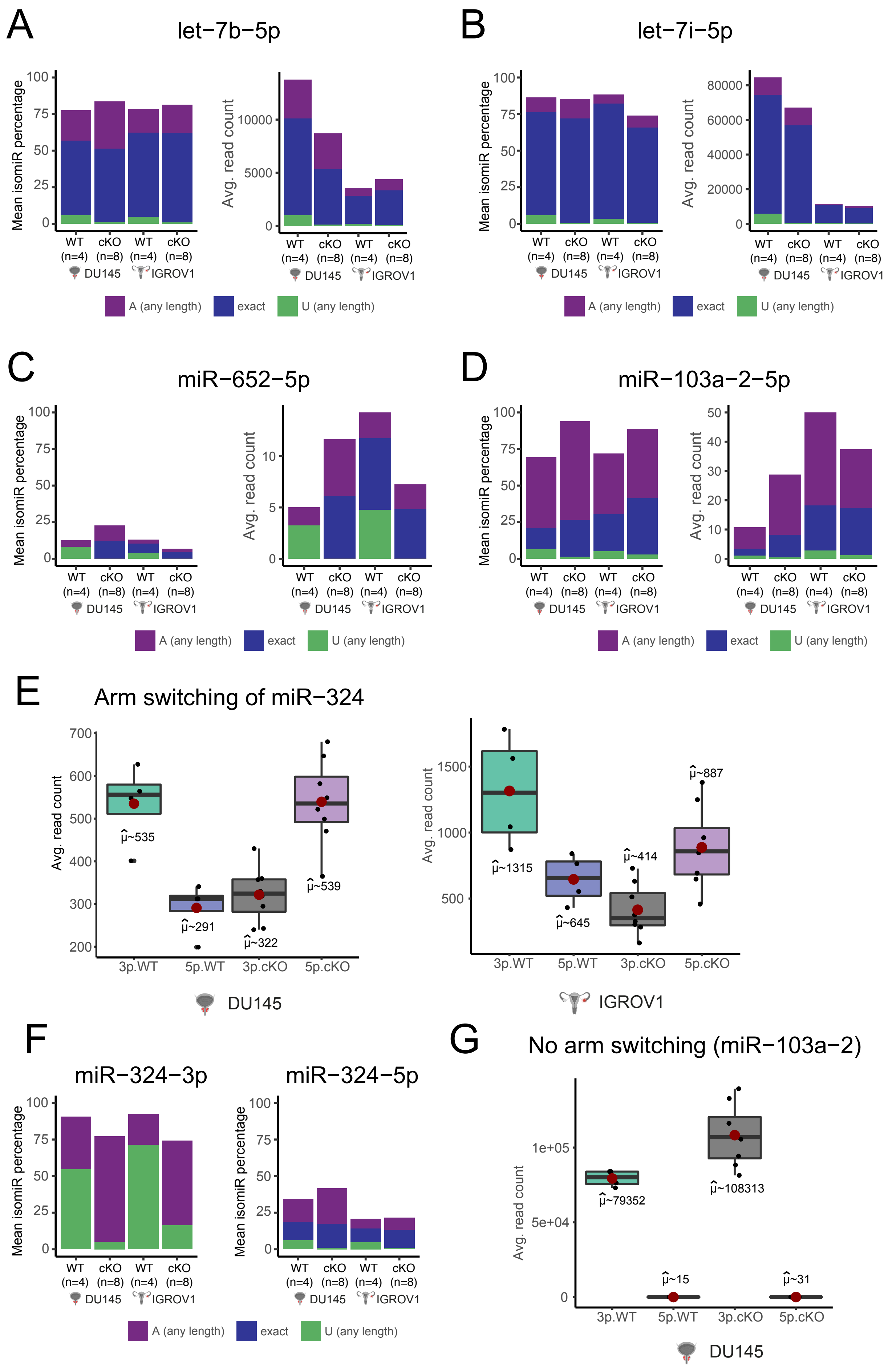
A
let−7b−5p
B
let−7i−5p
C
miR−652−5p
D
miR−103a−2−5p
Mean isomiR percentage
Avg. read count
WT (n=4)
cKO (n=8)
DU145
IGROV1
A (any length)
exact
U (any length)
E
Arm switching of miR−324
μ̂~535
μ̂~291
μ̂~322
μ̂~539
μ̂~1315
μ̂~645
μ̂~414
μ̂~887
3p.WT
5p.WT
3p.cKO
5p.cKO
F
miR−324−3p
miR−324−5p
G
No arm switching (miR−103a−2)
μ̂~79352
μ̂~15
μ̂~108313
μ̂~31

Figure S4

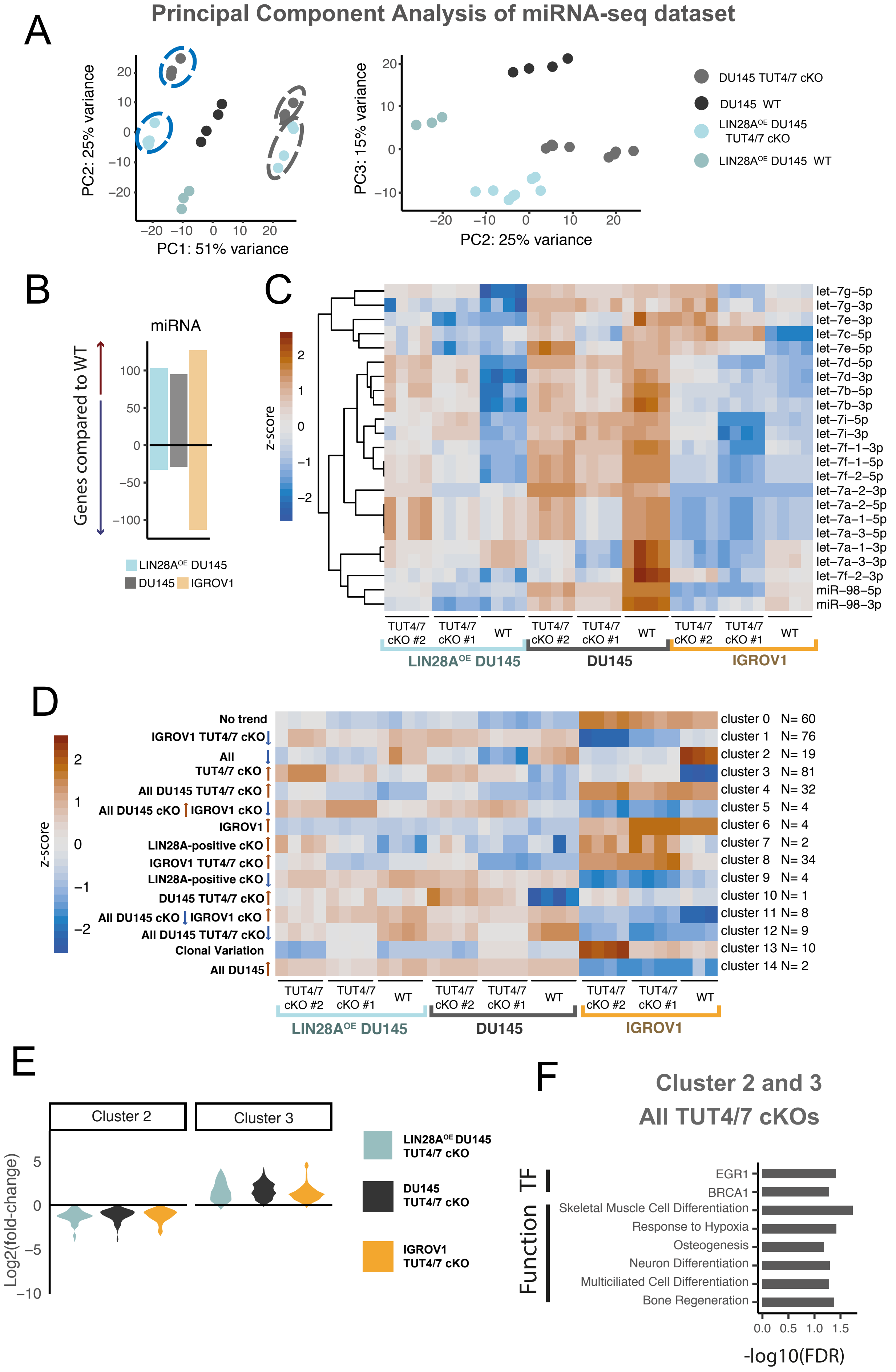

A
Principal Component Analysis of miRNA-seq dataset
PC2: 25% variance
PC1: 51% variance
PC3: 15% variance
PC2: 25% variance
DU145 TUT4/7 cKO
DU145 WT
LIN28A^OE DU145 TUT4/7 cKO
LIN28A^OE DU145 WT
B
miRNA
Genes compared to WT
LIN28A^OE DU145
DU145
IGROV1
C
z-score
let–7g–5p 2 +
let–7g–3p 2 +
let–7e–3p 1 -
let–7c–5p 1 -
let–7e–5p 1 -
let–7d–5p 2 +
let–7d–3p 2 +
let–7b–5p 2 +
let–7b–3p 2 +
let–7i–5p 2 +
let–7i–3p 2 +
let–7f–1–3p 2 +
let–7f–1–5p 2 +
let–7f–2–5p 2 -
let–7a–2–3p 1 -
let–7a–2–5p 1 -
let–7a–1–5p 2 -
let–7a–3–5p 2 -
let–7a–1–3p 2 -
let–7a–3–3p 2 -
let–7f–2–3p 2 -
miR–98–5p 2 +
miR–98–3p 2 +
TUT4/7 cKO #2
TUT4/7 cKO #1
WT
LIN28A^OE DU145
DU145
IGROV1
D
z-score
No trend
IGROV1 TUT4/7 cKO↓
All TUT4/7 cKO ↓↑
All DU145 TUT4/7 cKO↑
All DU145 cKO↑ IGROV1 cKO↓
IGROV1↑
LIN28A-positive cKO↑
IGROV1 TUT4/7 cKO↑
LIN28A-positive cKO↓
DU145 TUT4/7 cKO↑
All DU145 cKO↓ IGROV1 cKO↑
All DU145 TUT4/7 cKO↓
Clonal Variation
All DU145↑
cluster 0 N= 60
cluster 1 N= 76
cluster 2 N= 19
cluster 3 N= 81
cluster 4 N= 32
cluster 5 N= 4
cluster 6 N= 4
cluster 7 N= 2
cluster 8 N= 34
cluster 9 N= 4
cluster 10 N= 1
cluster 11 N= 8
cluster 12 N= 9
cluster 13 N= 10
cluster 14 N= 2
TUT4/7 cKO #2
TUT4/7 cKO #1
WT
LIN28A^OE DU145
DU145
IGROV1
E
Cluster 2
Cluster 3
Log2(fold-change)
LIN28A^OE DU145 TUT4/7 cKO
DU145 TUT4/7 cKO
IGROV1 TUT4/7 cKO
F
Cluster 2 and 3
All TUT4/7 cKOs
TF
EGR1
BRCA1
Function
Skeletal Muscle Cell Differentiation
Response to Hypoxia
Osteogenesis
Neuron Differentiation
Multiciliated Cell Differentiation
Bone Regeneration
-log10(FDR)

Figure S5

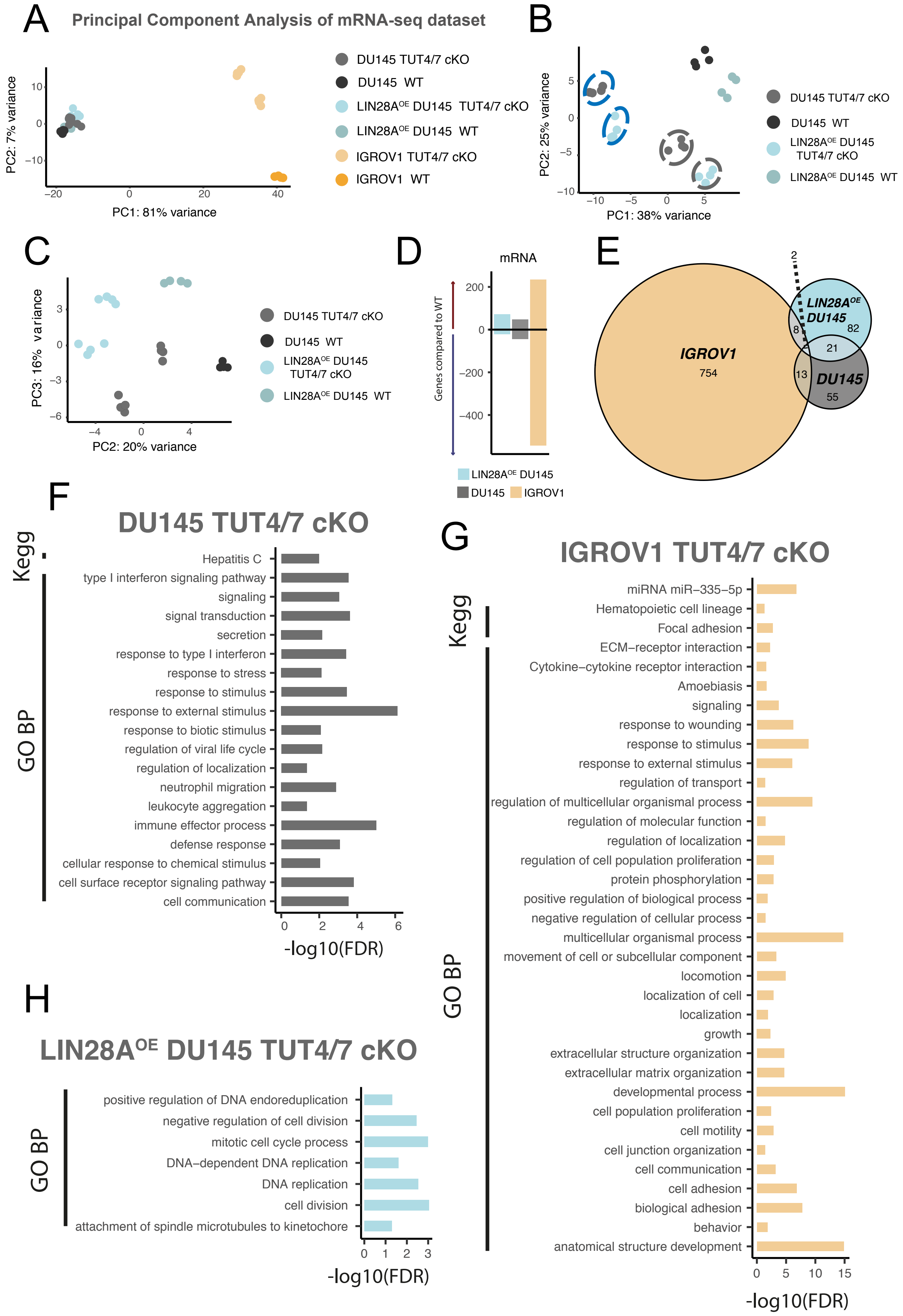

A
Principal Component Analysis of mRNA-seq dataset
PC2: 7% variance
PC1: 81% variance
DU145 TUT4/7 cKO
DU145 WT
LIN28A^OE DU145 TUT4/7 cKO
LIN28A^OE DU145 WT
IGROV1 TUT4/7 cKO
IGROV1 WT
B
PC2: 25% variance
PC1: 38% variance
DU145 TUT4/7 cKO
DU145 WT
LIN28A^OE DU145 TUT4/7 cKO
LIN28A^OE DU145 WT
C
PC3: 16% variance
PC2: 20% variance
DU145 TUT4/7 cKO
DU145 WT
LIN28A^OE DU145 TUT4/7 cKO
LIN28A^OE DU145 WT
D
mRNA
Genes compared to WT
LIN28A^OE DU145
DU145
IGROV1
E
IGROV1
754
LIN28A^OE DU145
82
DU145
55
2
8
21
13
F
DU145 TUT4/7 cKO
Kegg
GO BP
Hepatitis C
type I interferon signaling pathway
signaling
signal transduction
secretion
response to type I interferon
response to stress
response to stimulus
response to external stimulus
response to biotic stimulus
regulation of viral life cycle
regulation of localization
neutrophil migration
leukocyte aggregation
immune effector process
defense response
cellular response to chemical stimulus
cell surface receptor signaling pathway
cell communication
-log10(FDR)
G
IGROV1 TUT4/7 cKO
Kegg
GO BP
miRNA miR−335−5p
Hematopoietic cell lineage
Focal adhesion
ECM−receptor interaction
Cytokine−cytokine receptor interaction
Amoebiasis
signaling
response to wounding
response to stimulus
response to external stimulus
regulation of transport
regulation of multicellular organismal process
regulation of molecular function
regulation of localization
regulation of cell population proliferation
protein phosphorylation
positive regulation of biological process
negative regulation of cellular process
multicellular organismal process
movement of cell or subcellular component
locomotion
localization of cell
localization
growth
extracellular structure organization
extracellular matrix organization
developmental process
cell population proliferation
cell motility
cell junction organization
cell communication
cell adhesion
biological adhesion
behavior
anatomical structure development
-log10(FDR)
H
LIN28A^OE DU145 TUT4/7 cKO
GO BP
positive regulation of DNA endoreduplication
negative regulation of cell division
mitotic cell cycle process
DNA−dependent DNA replication
DNA replication
cell division
attachment of spindle microtubules to kinetochore
-log10(FDR)

Figure S6

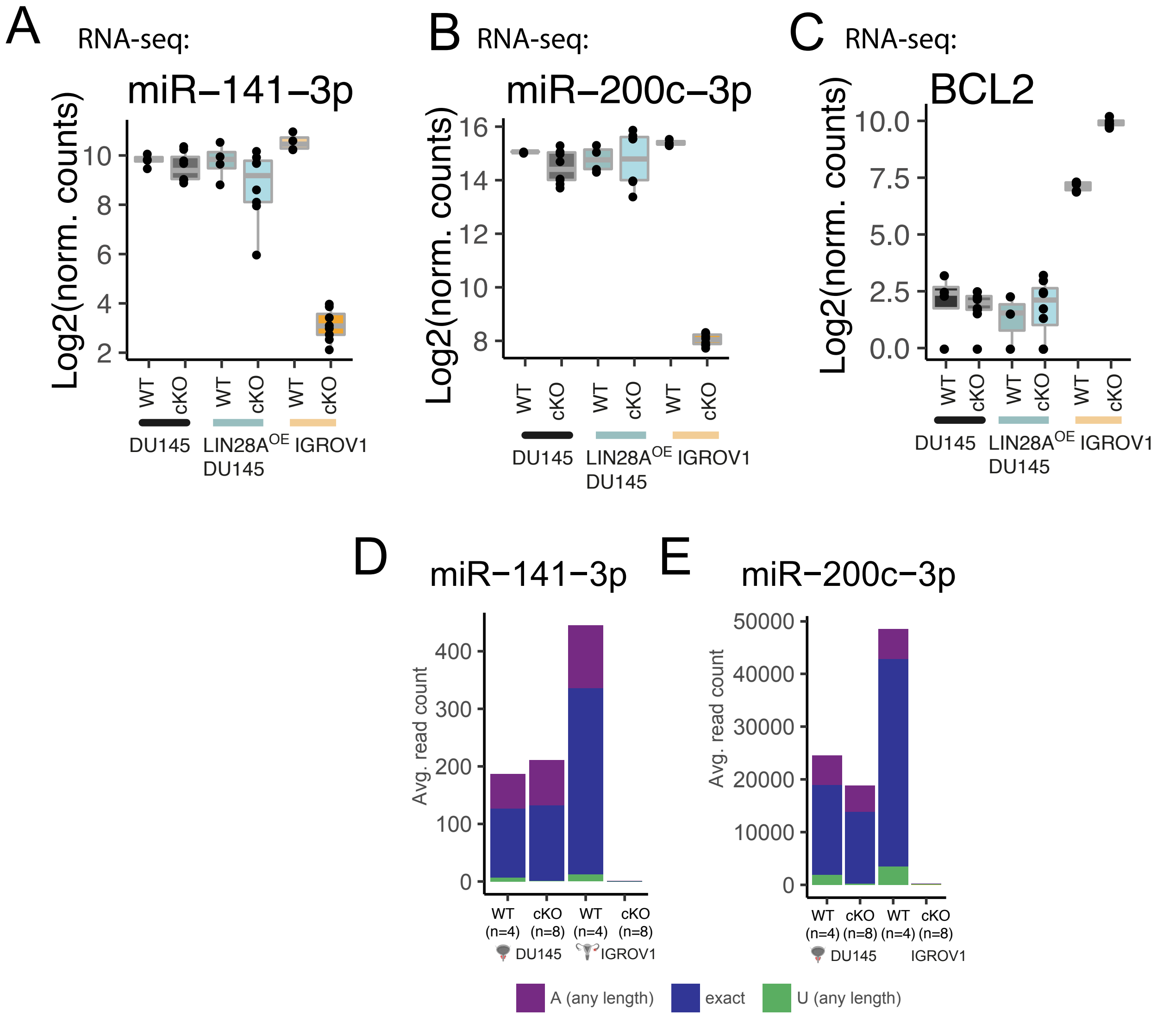

A RNA-seq:
miR−141−3p
Log2(norm. counts)
2 4 6 8 10
WT cKO WT cKO WT cKO
DU145 LIN28A^OE DU145 IGROV1
B RNA-seq:
miR−200c−3p
Log2(norm. counts)
8 10 12 14 16
WT cKO WT cKO WT cKO
DU145 LIN28A^OE DU145 IGROV1
C RNA-seq:
BCL2
Log2(norm. counts)
0.0 2.5 5.0 7.5 10.0
WT cKO WT cKO WT cKO
DU145 LIN28A^OE DU145 IGROV1
D
miR−141−3p
Avg. read count
0 100 200 300 400
WT (n=4) cKO (n=8) WT (n=4) cKO (n=8)
DU145 IGROV1
E
miR−200c−3p
Avg. read count
0 10000 20000 30000 40000 50000
WT (n=4) cKO (n=8) WT (n=4) cKO (n=8)
DU145 IGROV1
A (any length) exact U (any length)

Figure S7

A
Raw western blots for Figures 1A-D

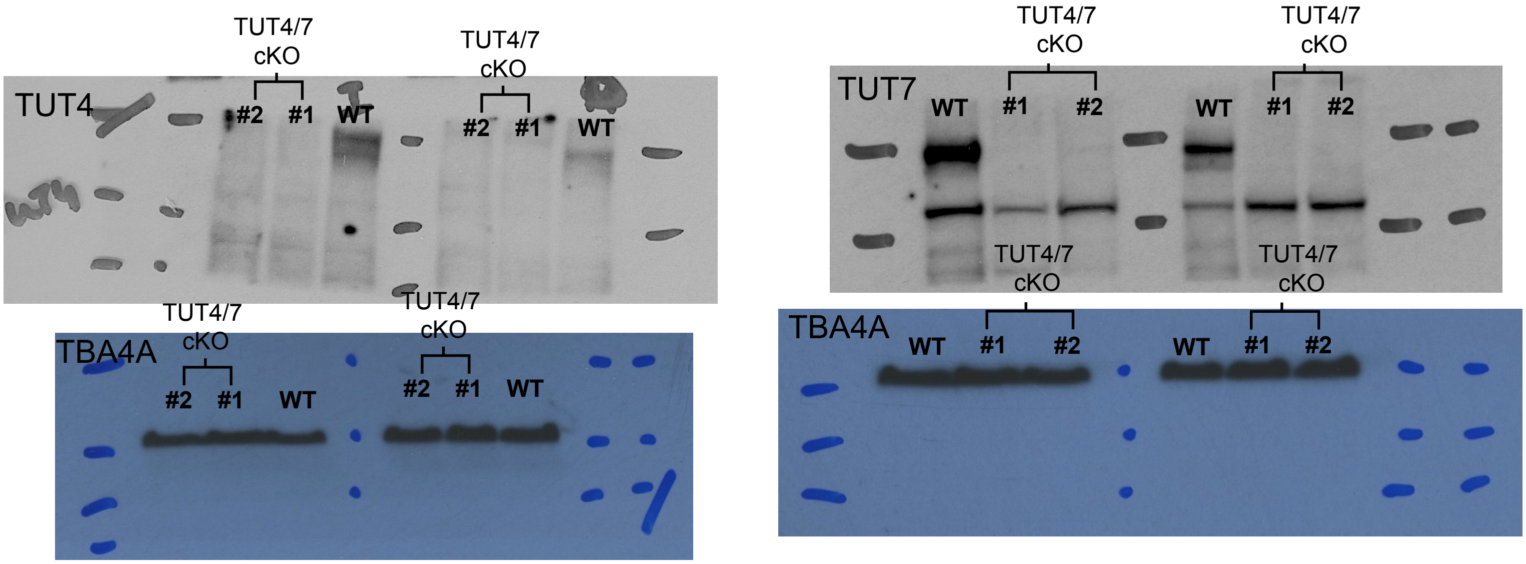

B
Raw western blots for Figures 4A

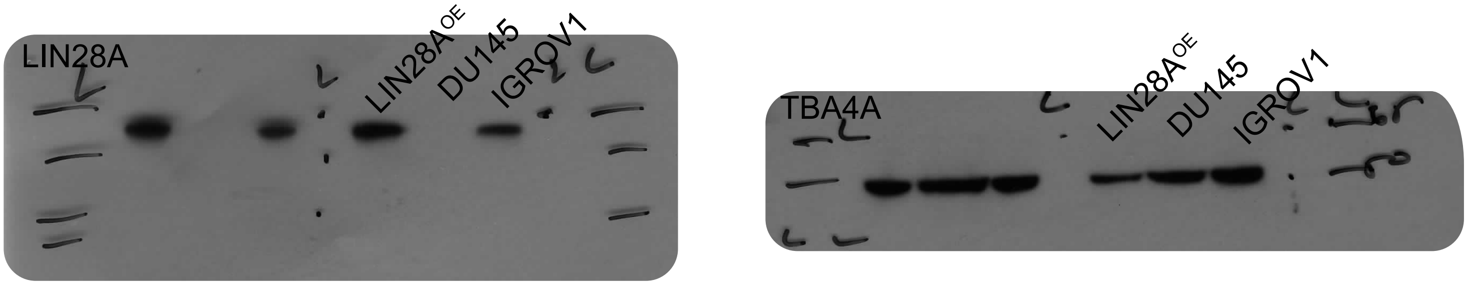

C

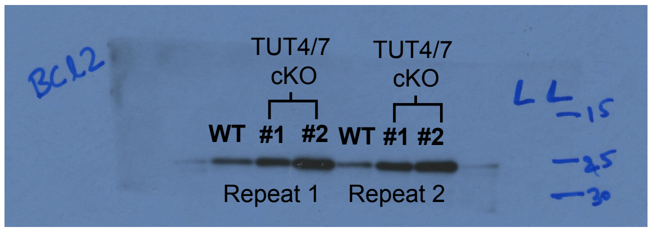

Raw western blot for BCL2 for Figure 5F

D

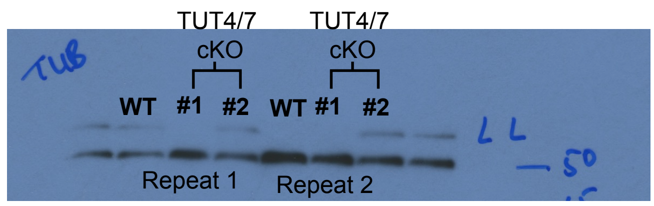

Raw western blot for TBA4A for Figure 5F
